# *Plasmodium* parasite exploits host aquaporin-3 during liver stage malaria infection

**DOI:** 10.1371/journal.ppat.1007057

**Published:** 2018-05-18

**Authors:** Dora Posfai, Kayla Sylvester, Anupama Reddy, Jack G. Ganley, Johannes Wirth, Quinlan E. Cullen, Tushar Dave, Nobutaka Kato, Sandeep S. Dave, Emily R. Derbyshire

**Affiliations:** 1 Department of Molecular Genetics and Microbiology, Duke University Medical Center, North Carolina, United States of America; 2 Department of Medicine, Duke University Medical Center, Durham, North Carolina, United States of America; 3 Department of Chemistry, Duke University, Durham, North Carolina, United States of America; 4 The Broad Institute, 7 Cambridge Center, Cambridge, Massachusetts, United States of America; University of Bern, SWITZERLAND

## Abstract

Within the liver a single *Plasmodium* parasite transforms into thousands of blood-infective forms to cause malaria. Here, we use RNA-sequencing to identify host genes that are upregulated upon *Plasmodium berghei* infection of hepatocytes with the hypothesis that host pathways are hijacked to benefit parasite development. We found that expression of aquaporin-3 (AQP3), a water and glycerol channel, is significantly induced in *Plasmodium-*infected hepatocytes compared to uninfected cells. This aquaglyceroporin localizes to the parasitophorous vacuole membrane, the compartmental interface between the host and pathogen, with a temporal pattern that correlates with the parasite’s expansion in the liver. Depletion or elimination of host AQP3 expression significantly reduces *P*. *berghei* parasite burden during the liver stage and chemical disruption by a known AQP3 inhibitor, auphen, reduces *P*. *falciparum* asexual blood stage and *P*. *berghei* liver stage parasite load. Further use of this inhibitor as a chemical probe suggests that AQP3-mediated nutrient transport is an important function for parasite development. This study reveals a previously unknown potential route for host-dependent nutrient acquisition by *Plasmodium* which was discovered by mapping the transcriptional changes that occur in hepatocytes throughout *P*. *berghei* infection. The dataset reported may be leveraged to identify additional host factors that are essential for *Plasmodium* liver stage infection and highlights *Plasmodium’s* dependence on host factors within hepatocytes.

## Author summary

*Plasmodium* parasites undergo an obligatory morphogenesis and replication within the liver before they invade red blood cells and cause malaria. The liver stage is clinically silent but essential for the *Plasmodium* parasite to complete its life cycle. During this time, the parasite relies on the host cell to support a massive replication event, yet host factors that are critical to this expansion are largely unknown. We identify human aquaporin-3 (AQP3), a water and glycerol channel, as essential for the proper development of the parasite within the liver cell. AQP3 localizes to the parasitophorous vacuole membrane, the interface between the host cytoplasm and the parasite, possibly aiding in the nutritional uptake for the parasite. Genetic disruption or treatment with the AQP3 inhibitor auphen, reduces parasite load in liver and blood cells.

## Introduction

Malaria remains one of the greatest burdens to global health with an estimated 440,000 deaths in 2015 [1]. While fatalities attributed to malaria have significantly declined over the past decade as a result of increased interventions, the spread of resistance to current antimalarial treatments and insecticides threatens the progress that has been made in eliminating this infectious disease. The causative agent of malaria is the *Plasmodium* parasite, an obligate intracellular pathogen that strategically exploits host processes to ensure its survival. Many of these interactions between the host and parasite remain unknown, particularly during the parasite’s elusive liver stage.

The *Plasmodium* parasite is transferred to the human host via the bite of an *Anopheles* mosquito in the form of a sporozoite. Before the *Plasmodium* parasite can invade erythrocytes and cause disease, it must first pass through an obligatory liver stage. Sporozoites travel through the bloodstream to the liver where they pass through multiple hepatocytes before establishing an infection in a final cell [2–5]. As the sporozoite invades the hepatocyte, invagination of the host cell membrane forms the parasitophorous vacuole membrane (PVM) around the parasite, protecting it from clearance [6, 7]. It is within this PV that the sporozoite undergoes dramatic morphological changes to become the exo-erythrocytic form (EEF) [8], which subsequently divides into tens of thousands of merozoites within a single hepatocyte [9]. After maturation, the blood infective merozoites are released into the blood stream where they cause the symptoms of malaria.

The liver stage of the *Plasmodium* life cycle is an attractive target for drug and vaccine development as it represents a bottleneck in the parasite population. Furthermore, inhibition of liver-stage parasites prevents disease manifestation that emerges in the subsequent blood stage. During this stage, the parasite relies on host cell factors for successful invasion, development, and nutrient acquisition to support its massive replication [10]. However, few host proteins that are involved in the maturation of these parasites have been identified. The host CD81 receptor [11], scavenger receptor type B class I (SR-BI) [12, 13], and the surface protein EphA2 [14] have all been identified as important invasion factors, but among these only SR-BI has been implicated in the maturation of parasites after invasion. Few other host proteins are known to be critical for the developing parasites. Thus, while significant advances have been made to our current understanding of hepatocyte invasion, host-pathogen interactions that are beneficial for the parasite’s development within the hepatocyte remain poorly understood. Host-based strategies represent an attractive, yet underexplored route to malaria control as they reduce the probability that parasites will develop drug resistance when compared to parasite-directed therapeutics [15]. However, a greater understanding of host-parasite interactions during the liver stage is critical to develop these measures.

A comprehensive analysis of the host response to infection during the liver stage has been challenging because of the low infection rate of hepatocytes by sporozoites [16] and the technical difficulty of obtaining sporozoites in large quantities from the salivary glands of live mosquitoes. Previous work aiming to elucidate important hepatocyte factors assessed the host transcriptome in response to *P*. *berghei* infection by microarray analysis. Researchers found an initial host response to stress followed by maintenance of cell viability in response to infection [17]. This important work provided a global analysis of the host response to *Plasmodium* infection, but it remains unclear whether any of the differentially expressed genes (DEGs) are regulated in a manner that is advantageous to the parasite. In addition to the previously reported sequential regulation of genes in hepatocytes, we hypothesized that conserved host-parasite interactions during the liver stage have evolved that are essential for the proper maturation and development of EEFs. To search for essential host factors, we used RNA-sequencing (RNA-seq) to identify genes that are induced in HepG2 hepatocytes upon *P*. *berghei* infection. Among genes that were differentially expressed, aquaporin-3 (AQP3) was chosen for functional analysis because it was one of the most highly induced genes upon infection and because of previous literature related to aquaporins and parasite development.

Aquaporins (AQPs) are a family of highly conserved membrane proteins that transport water and other small solutes [18]. There are 13 human AQP isoforms that belong to one of two groups: orthodox aquaporins that strictly transport water and aquaglyceroporins that are capable of transporting small solutes, such as glycerol, in addition to water molecules across membranes [19, 20]. The role of the aquaporin family of proteins has been previously studied in the context of *Plasmodium* infections, but primarily focusing of the parasite aquaglyceroporin [21]. The *P*. *falciparum* aquaporin, *Pf*AQP, localizes to the parasite cell membrane where it is thought to be critical for glycerol transport. The role of host aquaporins during *Plasmodium* infection is understood to a lesser extent, but have been implicated in blood stage infections [21] where one report demonstrated that AQP3 localizes to the parasitophorous vacuole of *P*. *falciparum* infected red blood cells [22].

AQP3 is an aquaglyceroporin that transports both water and glycerol in mammalian cells [20, 23]. We found that AQP3 expression was induced in human hepatocytes in response to *P*. *berghei* infection at times that implicated a role in the rapid expansion and replication of the parasite. Our subsequent molecular studies revealed that this host protein is actively trafficked to the PVM after invasion. Genetic disruption or depletion of AQP3 leads to a significant decrease in *P*. *berghei* liver stage parasite load. Furthermore, treatment with auphen, a known AQP3 inhibitor, reduces both *P*. *berghei* parasite load in hepatocytes and *P*. *falciparum* parasite load in erythrocytes, suggesting the host protein has an essential role during various parasite life stages. Importantly, deletion of AQP3 in mice is not lethal [24], suggesting the host protein has potential as a possible therapeutic target. This work enhances our current understanding of host liver processes influenced by *Plasmodium* infection and highlights the potential of targeting these processes for future drug and vaccine development against malaria. Further, the provided datasets of DEGs during *Plasmodium* infection can serve as the foundation for future studies aimed at understanding the host response.

## Results

### Screening of host gene expression implicates AQP3 in *Plasmodium* infection

To evaluate potential host responses to *Plasmodium* infection, we performed RNA-seq on HepG2 human hepatocytes infected with GFP-expressing *P*. *berghei*. Because of the inherently low infection rate of sporozoites in the best in vitro model systems [16], we isolated infected cells by fluorescence-activated cell sorting (FACS) (S1 Fig). Approximately 3,000 *Plasmodium* infected-cells were sorted at 4, 24, and 48 hpi as well as uninfected cells in 2–4 biological replicates. RNA isolation and library preparation followed by sequencing was achieved using a protocol designed for low cell numbers. From an evaluation of each candidate gene, AQP3 was chosen for functional studies because it was one of the most significantly induced genes and evidence from previous studies that aquaporins are involved in *Plasmodium* infection [21, 22].

### Host AQP3 is upregulated and incorporated into the parasitophorous vacuole membrane in response to infection

**Fig 1:**
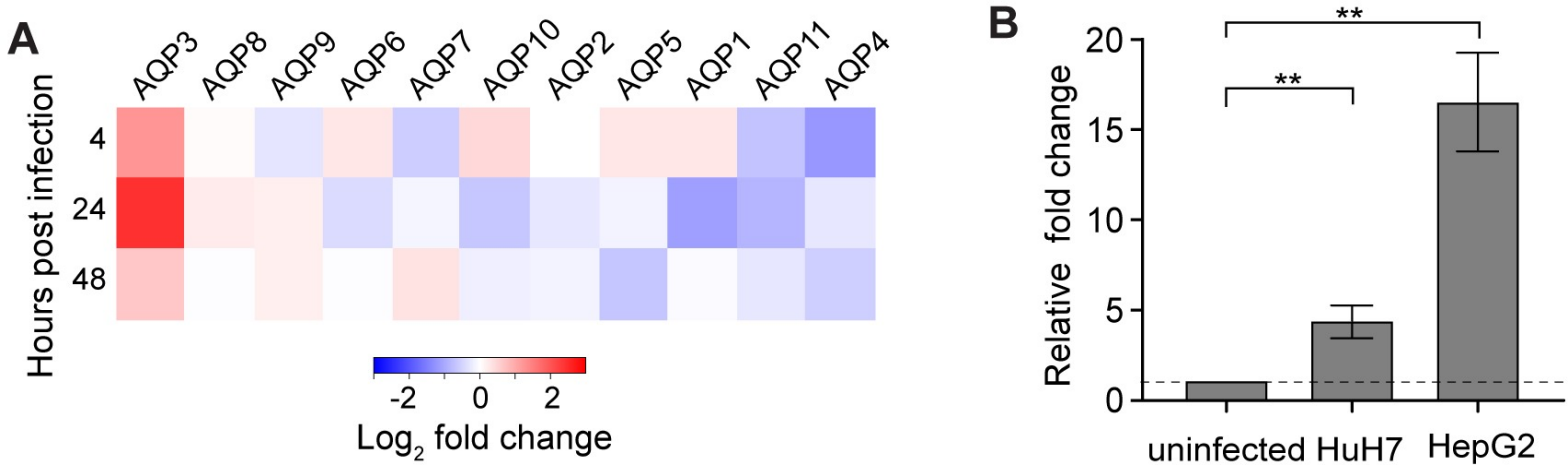
Differential gene expression of aquaporins during the liver stage of *P*. *berghei*. (A) Differential gene expression of all human aquaporin isoforms in HepG2 hepatocytes post *P*. *berghei* infection compared to uninfected cells (n = 2–4 biological replicates). AQP3 is differentially expressed (*p* = 1.55 x 10^−7^) in infected cells, but other aquaporin family members do not exhibit significant differential expression. (B) qRT-PCR quantification of AQP3 mRNA expression at 48 hpi in both HuH7 and HepG2 cells. *P*. *berghei* infected HuH7 cells had a 4.1-fold increase in AQP3 mRNA transcripts compared to uninfected HuH7 cells (**p = 0.0075, unpaired Student’s *t-*test; n = 4 independent experiments) and HepG2 infected cells had a 15-fold increase in AQP3 mRNA transcripts compared to uninfected HepG2 cells (**p = 0.001, unpaired Student’s *t-*test; n = 3 independent experiments). Error bars represent SEM.

RNA-seq analysis shows that AQP3 is one of the most differentially expressed genes in HepG2 cells infected with *P*. *berghei*. Human AQP3, which is widely expressed in the kidney, skin, and erythrocytes [25–27], can transport both water and glycerol. The human genome encodes thirteen AQP isoforms, and based on literature, AQP3, AQP6, AQP7, AQP8, AQP9, and AQP11 mRNAs are expressed in uninfected HepG2 cells [28]. Upon infection, AQP3 expression is induced and it is the only aquaporin isoform to be significantly (*p* = 1.55 x 10^−7^) differentially expressed (Fig 1A). We further confirmed the induction of AQP3 expression in *P*. *berghei*-infected hepatocytes by qRT-PCR of RNA extracted from both HepG2 and HuH7 infected cells (Fig 1B). At 48 hpi, HepG2 cells exhibited a 15-fold increase in AQP3 mRNA expression (*p* = 0.001, two-tailed Student’s *t*-test; n = 3 independent experiments) and HuH7 cells, an alternate human hepatoma cell line, had a 4-fold increase in AQP3 expression compared to uninfected HuH7 cells (*p* = 0.0075, two-tailed Student’s *t*-test; n = 4 independent experiments).

**Fig 2:**
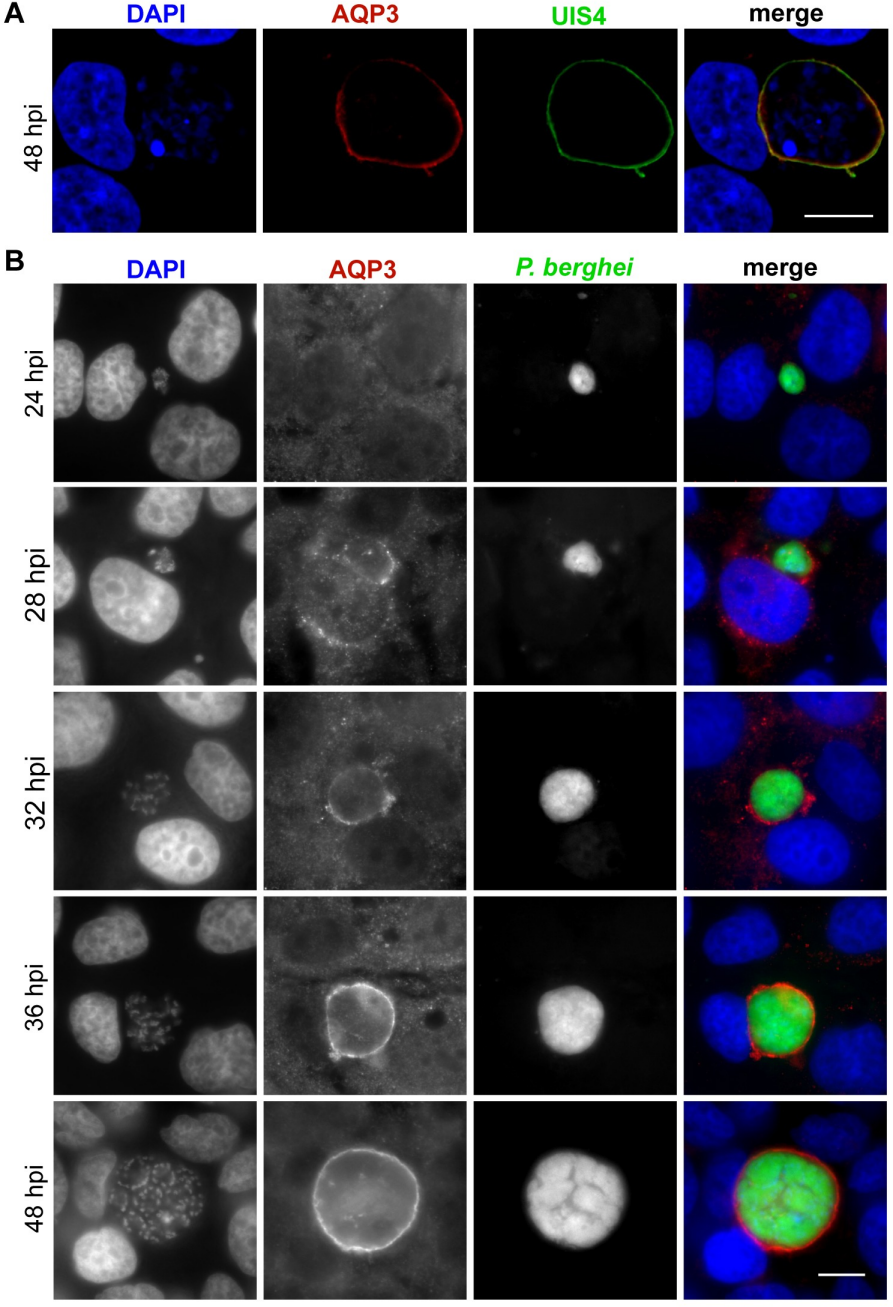
AQP3 localizes to the PVM in *P*. *berghei* infected hepatocytes. (A) Representative confocal image of a HepG2 cell infected with *P*. *berghei* and stained for UIS4 (green), *Hs*AQP3 (red), and DAPI (blue) at 48 hpi. Images represent 0.8 μm sections, scale bar = 10 μm. Pearson colocalization coefficient of 0.556 ± 0.014 (mean ± SD) calculated from the z-stacked images of three individual cells stained for *Hs*AQP3 and UIS4. (B) Widefield fluorescent microscopy of HuH7 cells infected with GFP-expressing *P*. *berghei* (green) and fixed at various times post infection. Cells were stained for *Hs*AQP3 (red) and DAPI (blue). Earliest localization of AQP3 to the PVM was detected at 28 hpi. After 32 hpi all EEFs have AQP3 staining. Scale bar, 10 μm.

AQP3 is a membrane protein that generally localizes to the plasma membrane in human cells where it facilitates the transport of water and glycerol [18]. However, upon hepatocyte infection by *Plasmodium* a second membrane is present, the PVM, which is derived from the host cell plasma membrane when the parasite first penetrates the hepatocyte. Because *Plasmodium* infection leads to the upregulation of AQP3 gene expression, we hypothesized that the protein localizes to the PVM of *Plasmodium-*infected hepatocytes. Evidence of human AQP3 localization to the PVM in *P*. *falciparum*-infected erythrocytes also supported this hypothesis [22]. To test if AQP3 localizes to the PVM during the liver stage, we used immunofluorescent (IF) microscopy with antibodies targeting human AQP3 and monitored the intracellular localization of the protein throughout infection. Visualization of AQP3 in *P*. *berghei-*infected hepatocytes demonstrated that the protein localizes to the PVM in both HepG2 and HuH7 cells (Fig 2, S1 Movie). We utilized confocal microscopy to show colocalization of AQP3 with UIS4, a parasite protein that is known to be integrated into the PVM [29], in HepG2 cells (Fig 2A). A Pearson colocalization coefficient of 0.56 ± 0.014 (mean ± SD) was calculated from z-stacked images of three individual cells with staining for AQP3 and UIS4. We also show that AQP3 localizes to the PVM of *P*. *berghei* infected HuH7 cells as early as 28 hpi, after the parasite initiates nuclear division (Fig 2B). Some infected cells showed partial localization of AQP3 to the host cell membrane at 28 hpi, but localization thereafter is restricted to the PVM. In this in vitro infection model system protein quantification by Western blot analysis is difficult due to the limited number of *Plasmodium*-infected cells that can be feasibly collected; however, antibody staining shows that protein levels are elevated in *P*. *berghei* infected hepatocytes when compared to uninfected cells (S2 Fig). Taken together, this analysis indicates that human AQP3 protein levels in hepatocytes increase in response to *P*. *berghei* infection, in agreement with the observed increase in gene transcription, and that AQP3 is actively trafficked to the PVM rather than being incorporated from the host cell membrane during parasite penetration of the host cell.

### Host AQP3 is essential for parasite development

**Fig 3:**
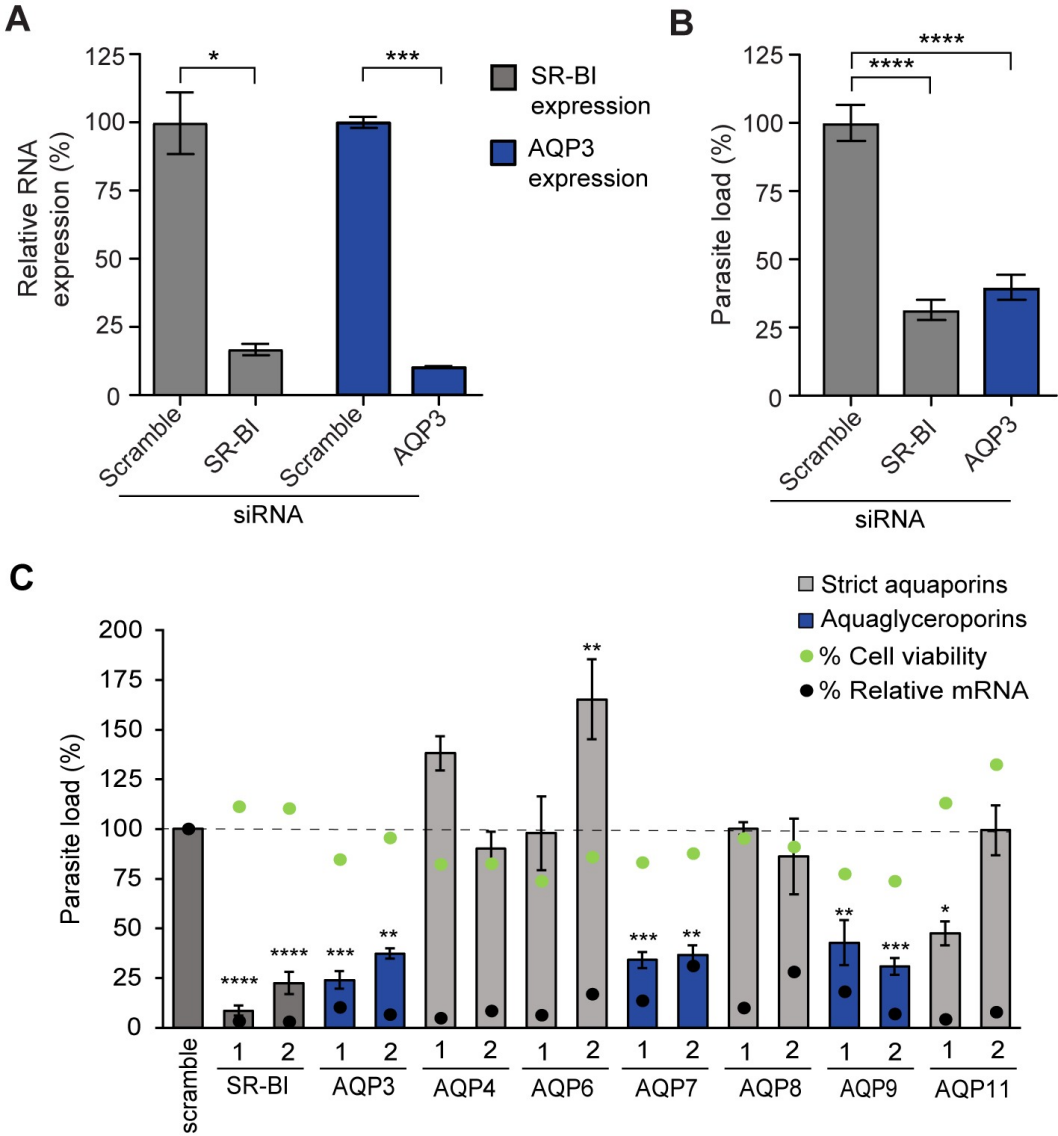
Depletion of aquaglyceroporins reduces parasite load in *P*. *berghei* infected HuH7 cells. (A) Relative mRNA expression of cells that were targeted by pooled AQP3 or SR-BI siRNAs at 50 nM. Reverse transfection of HuH7 cells by two siRNAs targeting AQP3 led to a 90% decrease in AQP3 mRNA transcripts (*p =* 0.005, unpaired Student’s *t*-test; n = 3 independent experiments), similar to the 86% efficiency in depletion of SR-BI, the positive control (*p =* 0.0186, unpaired Student’s *t*-test; n = 3 independent experiments). Genes were normalized to *Hs*18S RNA expression and compared to cells treated with non-targeting scrambled siRNAs. (B) siRNA depletion of AQP3 and SR-BI mRNA decreased parasite load by 60% and 68%, respectively, as measured by luminescence 48 hpi (*p* < 0.0001, One-way ANOVA, Dunnett’s multiple comparison test; n = 3 independent experiments). (C) siRNA depletion of all aquaporins expressed in hepatocytes, normalized to cells treated with non-targeting scramble siRNA. Bars represent the parasite load of cells treated with control siRNAs (dark grey), siRNAs targeting aquaglyceroporins (blue), and siRNAs targeting orthodox aquaporins (light grey) (One-way ANOVA, Dunnett’s multiple test comparison; n = 3–4 independent experiments). Green dots represent cell viability of these cells 48 hpi. Black dots represent the percent reduction in mRNA for the targeted genes as quantified by qRT-PCR 48 hours post transfection from one biological replicate. mRNA depletion of all aquaglyceroporins led to a significant reduction in *P*. *berghei* parasite load. A single siRNA targeting AQP11, an orthodox aquaporin, reduced the parasite load significantly. *p < 0.05, **p < 0.01, ***p < 0.001, ****p < 0.0001.

To address whether host AQP3 expression in *P*. *berghei* infected cells is necessary for the parasites to undergo morphological changes and replication within the PV, AQP3 mRNA was depleted by RNA interference (RNAi) and mutant AQP3 cell lines were generated. First, AQP3 mRNA expression was depleted by pooling two small interfering RNAs (siRNAs) targeting AQP3 (Qiagen). Scavenger receptor type B class I (SR-BI) siRNA depletion was used as a positive control as it is one of the few host genes that is known to be important for parasite development [12]. Efficiency of siRNA knockdowns was evaluated by extracting RNA from cells 48 hours after siRNA transfection and measuring mRNA expression of targeted genes by qRT-PCR. In parallel experiments, the impact of gene silencing on parasite load and cell viability was assessed in hepatocytes 48 hours after *P*. *berghei* infection. Parasite load after infection was quantified using a luciferase reporter constitutively expressed in *P*. *berghei* parasites [30]. Upon AQP3 siRNA reverse transfection of HuH7 cells, a 90% reduction in AQP3 transcripts resulted in a 60% reduction in parasite load (*p* < 0.0001, two-tailed Student’s *t*-test; n = 3 independent experiments) (Fig 3A and 3B). Reverse transfection of hepatocytes did not lead to any changes in cell viability 48 hpi (S3A Fig). The depletion of SR-BI transcripts by 86% led to a 68% reduction in parasite load (*p* < 0.0001, two-tailed Student’s *t*-test; n = 3 independent experiments), similar to what was observed in AQP3 depleted cells.

To further assess the impact of aquaporins on liver stage *Plasmodium* parasite development, all human aquaporin isoforms present in hepatocytes [28] were targeted by RNAi. Each gene was targeted by four individual siRNAs (S1 Table). Efficiency of siRNA knockdowns, parasite load, and cell viability were assessed (S2 Table, S3B and S3C Fig) and Fig 3 shows representative data that include the two siRNAs for each gene that correspond to the greatest mRNA transcript depletion. The only aquaporin genes with a significant decrease in expression and simultaneous reduction in parasite load, with a minimum of two separate siRNAs, were AQP3, AQP7 and AQP9, all of which are aquaglyceroporins. AQP3 mRNA depletion had the greatest influence on parasite load with an average of 69% reduction (Fig 3C). AQP7 and AQP9 depletion also significantly reduced parasite load, resulting in a 65% and 63% reduction on average, respectively (Fig 3C). One siRNA targeting the orthodox aquaporin, AQP11 (AQP11-1 in Fig 3C), reduced parasite load by 52% (*p* = 0.0343, One-way ANOVA, Dunnnet’s multiple comparison test). However, all four siRNAs reduced AQP11 transcripts (>85%) with only AQP11-1 significantly altering parasite load, suggesting its activity is due to off-target effects. Importantly, liver cell viability was not altered upon siRNA transfection of the selected aquaglyceroporins, indicating that parasite load reduction was not a byproduct of hepatocyte toxicity (*p* > 0.5, One-way ANOVA, Dunnett’s multiple comparison test; n = 3).

**Fig 4:**
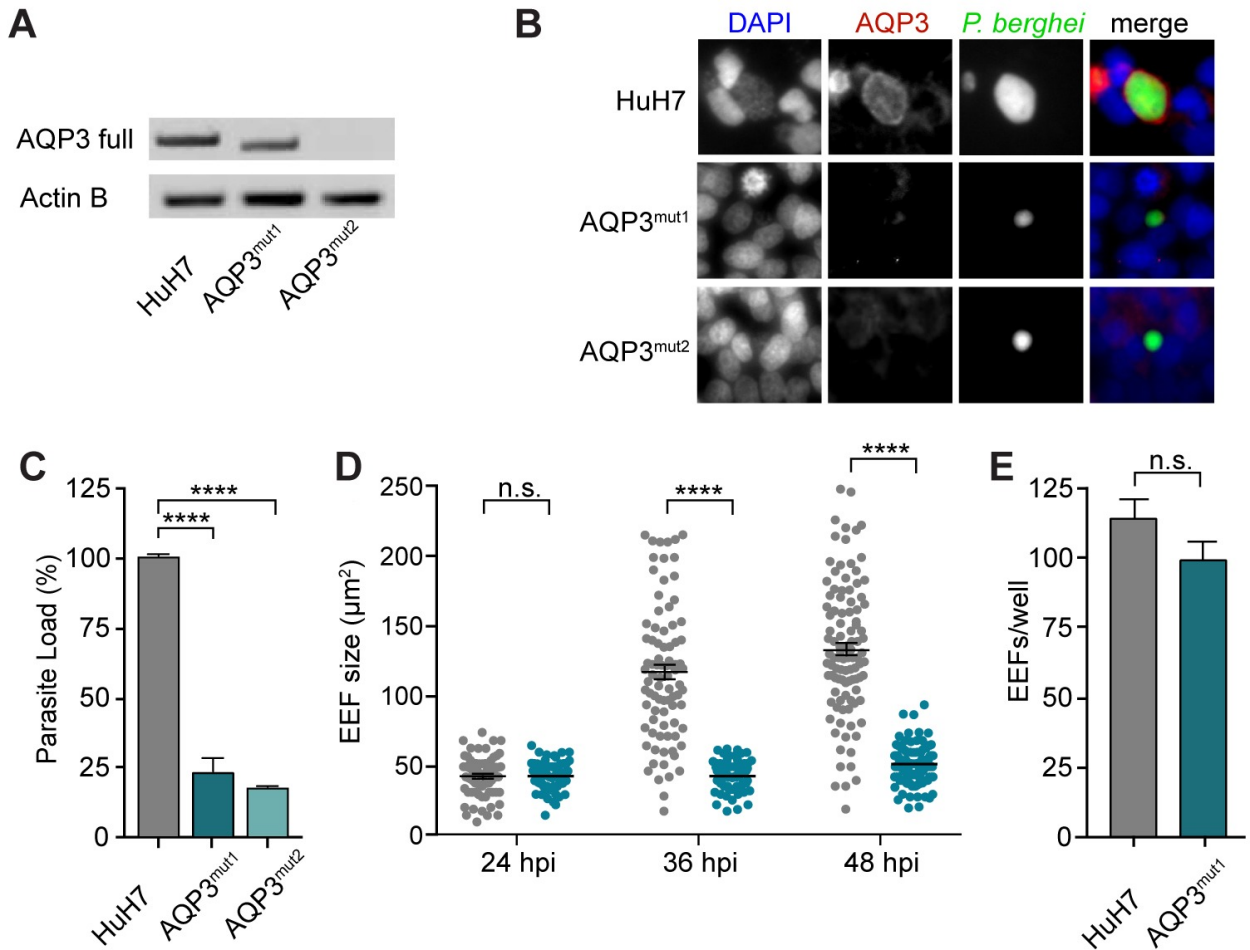
AQP3^mut^ cells prevent the proper development of *P*. *berghei* exo-erythrocytic forms. (A) Full length AQP3 transcript PCR amplification of RNA collected from wild type HuH7 cells and AQP3 mutated cell lines produced by CRISPR/Cas9 genome editing, AQP3^mut1^ and AQP3^mut2^. A truncated AQP3 transcript was amplified in AQP3^mut1^ while AQP3^mut2^ did not produce any AQP3 mRNA. (B) AQP3 protein was not detected by immunostaining in AQP3^mut1^ or AQP3^mut2^ cell lines at 48 hpi. Images of cells infected with *P*. *berghei* and stained for *Pb*Hsp70 (green), *Hs*AQP3 (red), and DAPI (blue). (C) Parasite load in AQP3^mut1^ and AQP3^mut2^ cells was significantly decreased by 77 ± 4.7% and 82 ± 0.24%, respectively (mean ± SEM; n = 3 independent experiments) (One-way ANOVA, Dunnett’s multiple comparison test). (D) Size of EEFs in HuH7 wild type cells (*grey*) and AQP3^mut1^ cells (*blue*) at 24, 36, and 48 hpi. EEF size was not significantly different at 24 hpi but was 65% and 64% smaller at 36 and 48 hpi, respectively, in AQP3^mut1^ compared to wild type cells (unpaired Student’s t-test; n > 100). (E) Number of EEFs was not significantly different in AQP3^mut1^ cells compared to wild type cells (*p =* 0.165, unpaired Student’s *t-*test; n = 5). ***p < 0.001 and ****p < 0.0001.

To validate the role of AQP3 on parasite load by an alternate means, the AQP3 gene was genetically mutated in HuH7 cells using CRISPR/Cas9 [31]. Multiple guide RNAs (gRNAs) were used for the generation of AQP3 mutant cell lines, targeting either exon 1 or 2 of the AQP3 gene (S3 Table). After single cell sorting, PCR amplification of the AQP3 gene in clonal populations was used to screen for mutations in the genomic DNA of the cells, resulting in four AQP3 mutant cell lines (AQP3^mut1-4^). All four AQP3^mut^ cell lines had confirmed mutations in the AQP3 gene and reduced *P*. *berghei* parasite load by approximately 80% compared to wild type HuH7 cells at 48 hpi (S4A Fig). Fig 4 shows the data for two of these AQP3^mut^ cell lines. Two gRNAs targeting exon 2 of the gene were used to generate AQP3^mut1^ and resulted in a 39 bp deletion (S4B and S4C Fig). Because the mutation did not result in a frame-shift, mRNA was still transcribed, however, protein was not detected by IF staining (Fig 4B), likely because of the inability of the protein to fold properly. Protein structure modeling of AQP3^mut1^ (SWISS-MODEL) suggests that the 39 bp deletion results in a nonfunctional channel that cannot transport nutrients (S4D Fig). Separately, AQP3^mut2^ was generated with two gRNAs targeting exon 1 of AQP3. mRNA transcripts and protein expression were not detected in this mutant cell line (Fig 4A and 4B). AQP3^mut1^ and AQP3^mut2^ cells had significantly lower parasite load 48 hours after *P*. *berghei* infection, with 77 and 82% reduction in parasite load, respectively, compared to wild type HuH7 cells (*p* < 0.001, One-way ANOVA, Dunnet’s multiple comparison test; n = 3 independent experiments) (Fig 4C). While genetic disruption of AQP3 significantly decreased parasite load in the mutant hepatocytes, it did not influence hepatocyte viability or cell growth (S4E Fig).

The EEFs after genetic disruption of AQP3 in liver cells were analyzed by microscopy for possible defects in development. EEF size calculations were compeleted by widefield fluorescent microscopy of HuH7 infected cells. The size of the EEFs in wild type HuH7 cells and AQP3^mut1^ cells were measured at 24, 36, and 48 hpi. Significant changes in size were not detected at 24 hpi with both cell lines having roughly 40 μm^2^ EEFs on average (Fig 4D) (*p* = 0.81, unpaired Student’s *t*-test; n > 70). However, by 36 hpi EEFs in wild type HuH7 cells were 2.9x larger than in AQP3^mut1^ cells, with EEFs averaging 112 and 40 μm^2^, respectively (*p* < 0.0001, unpaired Student’s *t*-test; n > 85). At 48 hpi EEFs in wild type HuH7 cells were 123 μm^2^ while EEFs in AQP3^mut1^ were 48 μm^2^ (*p* < 0.0001, unpaired Student’s *t*-test; n > 100). Interestingly, EEFs in AQP3^mut1^ cells had no significant increase in size between 24 and 36 hpi and only had an 18% increase in size between 24 and 48 hpi. For comparison, EEFs in wild type HuH7 had a 218% increase in size between 24 and 48 hpi. Thus, parasite growth is stunted during the late liver stage development of *P*. *berghei*. While the size of EEFs was significantly reduced in AQP3^mut1^ cells, the infection rate, measured by EEFs/well, was not altered (*p* = 0.165, two-tailed Student’s *t*-test) (Fig 4E). Three independent experiments were performed and representative data is shown from a single experiment. Significant changes were not observed in any experiments.

### AQP3 inhibitor auphen hinders parasite development at multiple stages of *Plasmodium* life cycle

**Fig 5:**
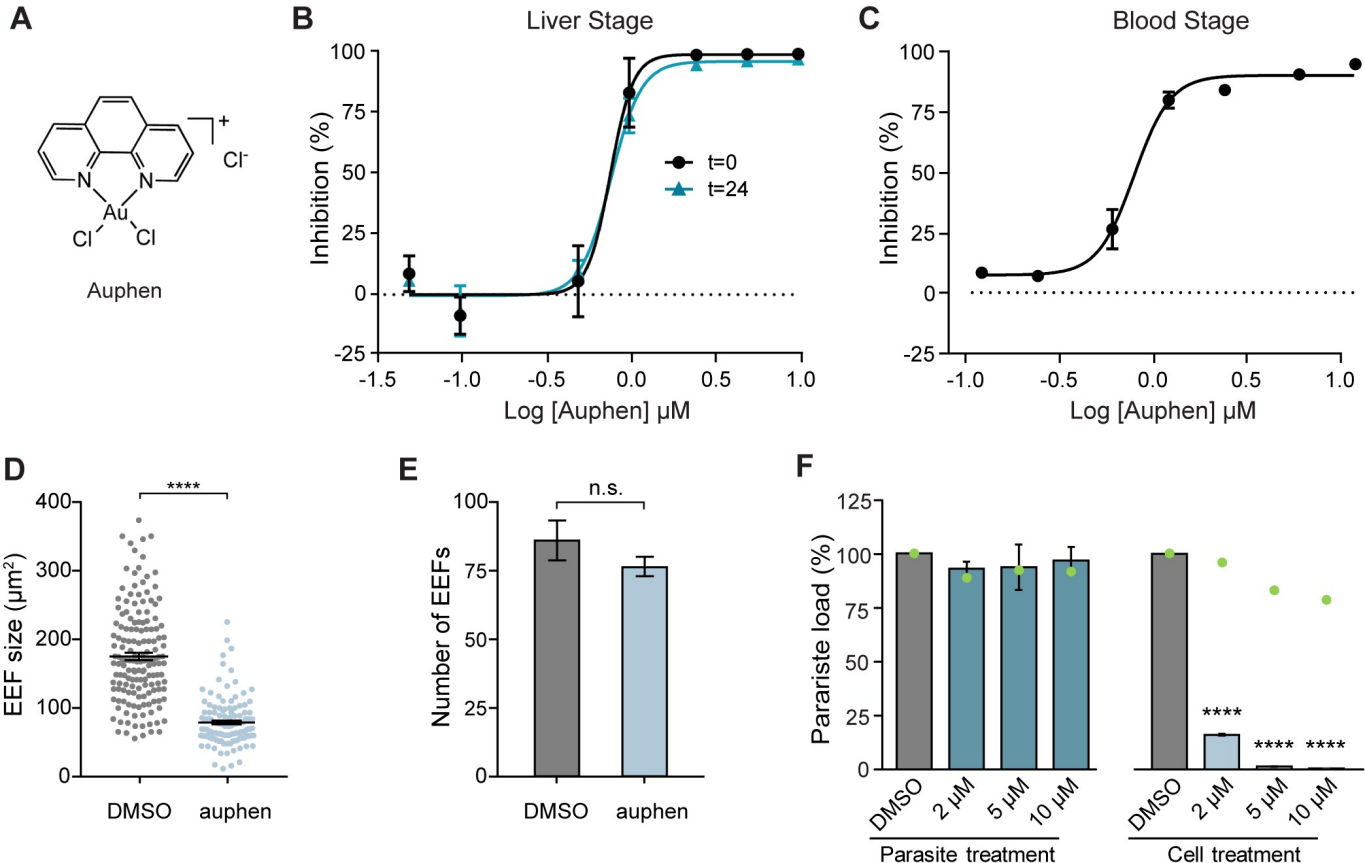
Auphen inhibits *Plasmodium* parasite development. (A) Chemical structure of auphen. (B) Parasite load of *P*. *berghei*-infected HuH7 cells measured at 48 hpi. Cells treated with auphen at time of infection (t = 0) exhibited an EC_50_ = 0.77 (n = 3 independent experiments) and cells treated with auphen at t = 24 had an EC_50_ = 0.78 μM (n = 1 independent experiment). (C) Parasite load of *P*. *falciparum* Dd2 infected erythrocytes treated with auphen at the time of infection, EC_50_ = 0.81 ± 0.10 μM (mean ± SEM; n = 2 independent experiments). (D) Size of EEFs in HuH7 cells infected with *P*. *berghei* and treated with DMSO compared to HuH7 cells treated with 1.2 μM auphen. HuH7 cells treated with auphen developed EEFs 56 ± 0.08% smaller than DMSO treated cells (*p* < 0.0001, unpaired Student’s *t-*test). (E) Number of EEFs was not significantly altered upon treatment with 1.2 μM auphen (*p =* 0.198, unpaired Student’s *t-*test; n = 9). (F) Parasite load of HuH7 cells infected with *P*. *berghei* sporozoites pre-incubated with DMSO, or various concentrations of auphen for 30 minutes prior to infection. Auphen treatment of sporozoites did not significantly reduce parasite load when measured 48 hpi (*p* = 0.9257, One-way ANOVA, Dunnett’s multiple comparison test; n = 2–3 independent experiments) while cells treated with auphen at the time of infection had significant reduction in parasite load (*p* < 0.0001, One-way ANOVA, Dunnett’s multiple comparison test; n = 2–3 independent experiments). ****p < 0.0001.

To probe if AQP3 is necessary for water permeability or glycerol transport we employed the gold-based compound [AuCl_2_(phen)]Cl (auphen) (Fig 5A) that selectively and irreversibly inhibits glycerol transport [32], but only minimally affects water permeability of AQP3 at concentrations above 100 μM [32, 33]. This functional selectivity is thought to be due to the interaction of Au(III) with a conserved cysteine residue at the selectivity filter of AQP3 that is not present in orthodox aquaporins [33]. Auphen was synthesized (S5 Fig) and found to inhibit liver stage parasite load with an EC_50_ = 0.77 ± 0.08 μM (mean ± SEM; n = 3 independent experiments) (Fig 5B), when *P*. *berghei* infected HuH7 hepatocytes were treated at 0 hpi. Because of the abundance of AQP3 in erythrocytes [27], we also tested auphen inhibition of *P*. *falciparum* Dd2-infected erythrocytes. Auphen treatment resulted in inhibition of *P*. *falciparum* with an EC_50_ of 0.81 ± 0.10 μM (mean ± SEM; n = 2) (Fig 5C), indicating that auphen is an effective inhibitor of multiple species and stages of the *Plasmodium* life cycle.

We hypothesized that auphen inhibits parasite load during the liver stage by inhibiting the glycerol permeability of AQP3. Most parasite functions that require glycerol, such as membrane and organelle synthesis, occur after 24 hpi, so we compared auphen potency with treatment at 0 and 24 hpi. Auphen treatment at 24 hpi yielded nearly identical EC_50_ values in both HepG2 and HuH7 cells when compared to cells treated at 0 hpi (Figs 5B and S6A). Importantly, hepatocyte cell viability was not affected by auphen treatment up to 20 μM (S6B Fig) (*p* = 0.165, One-Way ANOVA; n = 3 independent experiments). We also wanted to examine the effects of auphen when only treating cells during the early liver stage. HuH7 cells were treated with auphen (0.05–20 μM) at the time of infection and parasite load was quantified at 11 hpi, before the parasites begin to replicate, as well as after replication starts at 24 and 44 hpi (S6C Fig). Parasite load was not found to be significantly inhibited when assessed at 11 hpi (One-Way ANOVA, Tukey’s post hoc analysis, *p* > 0.05) even with 20 μM auphen treatment–the highest concentration tested. This observation indicates that auphen does not have inhibitory effects during the early stages of liver stage development. Additionally, by comparing the parasite load from early intrahepatic development (11 hpi) to late development (44 hpi) we observe that with high concentrations of auphen, parasite load is greater at early times when compared to later time points. This observation suggests that parasites that are unable to develop upon auphen treatment are cleared by the host cell. When the parasite load of auphen-treated cells was assessed at 24 hpi, inhibition was observed at 10 and 20 μM auphen, but this inhibition was substantially less than that observed at 44 hpi (S6C Fig). Taken together, this time course study indicates auphen acts between 12 and 48 hpi to inhibit *Plasmodium*.

The auphen mode of action was further assessed through a phenotypic study. Similar to AQP3^mut^ cells, auphen significantly reduced the size of EEFs in hepatocytes compared to control DMSO-treated hepatocytes (Fig 5D). HuH7 cells treated with 1.2 μM auphen developed EEFs 56 ± 0.08% smaller than DMSO-treated cells (*p* < 0.0001, unpaired Student’s *t-*test; n>100). Furthermore, the number of EEFs in cells treated with 1.2 μM auphen was not significantly different from that of wild type cells (Fig 5E) (*p =* 0.198, two-tailed Student’s *t-*test; n = 9). Additionally, to probe whether auphen acts on the host AQP3 and/or the parasite aquaporin, we pre-treated sporozoites with auphen prior to infection. Freshly dissected sporozoites were incubated with various concentrations of auphen or DMSO for 30 minutes. After the incubation, the sporozoites were pelleted and resuspended in media before being used to infect HuH7 cells. Treatment of sporozoites with 2–10 μM auphen did not significantly reduce parasite load in HuH7 cells when assessed at 48 hpi (*p* = 0.926, One-Way ANOVA; n = 2–3 independent experiments) while treating cells with the corresponding concentration of auphen immediately after infection reduced parasite load by 83.9–99.5% (*p* < 0.0001, One-Way ANOVA; n = 2–3 independent experiments) (Fig 5F). Pretreatment of hepatocytes with auphen also does not result in parasite inhibition (S6D Fig), which was expected as host AQP3 is not significantly expressed until after *P*. *berghei* infection.

**Fig 6:**
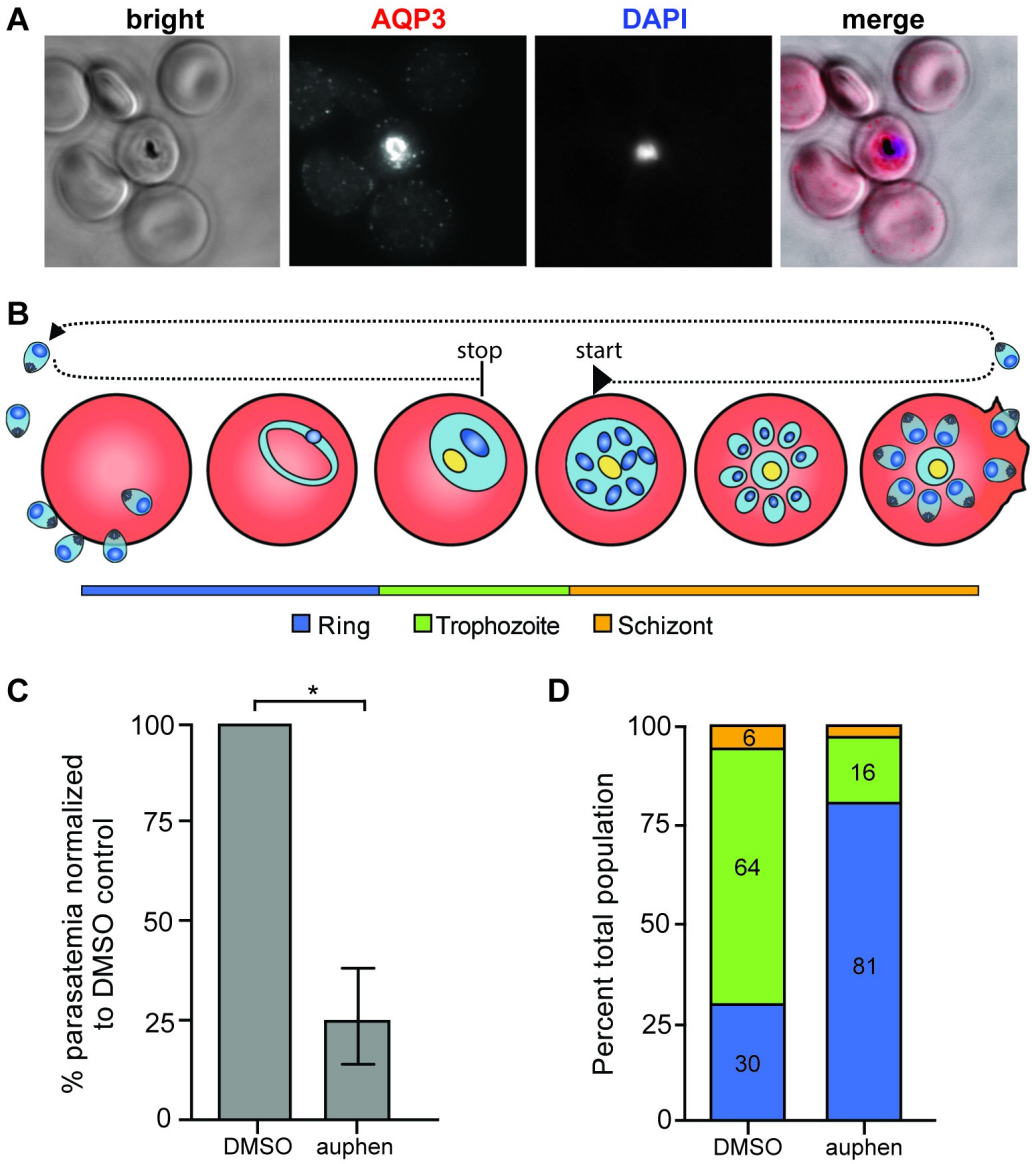
AQP3 localizes to the PVM and auphen treatment delays *P*. *falciparum* development in erythrocytes. (A) Fluorescent microscopy of erythrocytes infected with *P*. *falciparum* 3D7. Cells were stained with *Hs*AQP3 (red) and Hoechst (blue). AQP3 localizes to the PV of infected cells. (B) Schematic for auphen treatment of *P*. *falciparum* infected erythrocytes. Cells were treated with 2 μM auphen at the early schizont stage for 40 hours. (C) Parasitemia in auphen-treated cultures decreased 75 ± 12% compared to DMSO treated *P*. *falciparum-*infected erythrocytes (*p = 0.0257, unpaired Student’s *t-*test; n = 3 independent experiments). (D) After 40 hours of auphen treatment parasites were scored as ring, trophozoite, or schizont parasites. Auphen-treated *P*. *falciparum* exhibited a greater population of ring stage parasites when compared to the DMSO-treated control (n = 1 independent experiment).

The effects of auphen on blood-stage *Plasmodium* parasites was further evaluated. Immunofluorescent staining of AQP3 in *P*. *falciparum-*infected erythrocytes was completed to confirm the localization of the protein to the PVM as previously described [22]. We found that AQP3 localizes to the erythrocyte membrane in uninfected cells, but is translocated to the PVM of *P*. *falciparum*-infected cells (Fig 6A). Infected erythrocytes were visualized after treatment with 2 μM auphen or DMSO for 40 hours (Fig 6B). Treatment began during the early schizont stage to assess the ability of the parasites to complete maturation and invade new erythrocytes. Across three independent experiments, parasitemia significantly decreased by 75 ± 12% when treated with 2 μM auphen (Fig 6C) compared to DMSO treated erythrocytes (*p* = 0.0257, unpaired Student’s *t-*test). Erythrocyte toxicity was not observed microscopically in response to auphen treatment. For one biological replicate of *P*. *falciparum* auphen treatment, the parasite stages (ring, trophozoite, schizont) were scored to assess their development in the newly invaded erythrocytes (Fig 6D). Interestingly, the parasites that were able to infect erythrocytes after auphen treatment exhibited a developmental delay compared to the DMSO control as indicated by an increase in ring stage parasites and a decrease in trophozoites.

### Transcriptome analysis of *P*. *berghei*-infected hepatocytes

**Fig 7:**
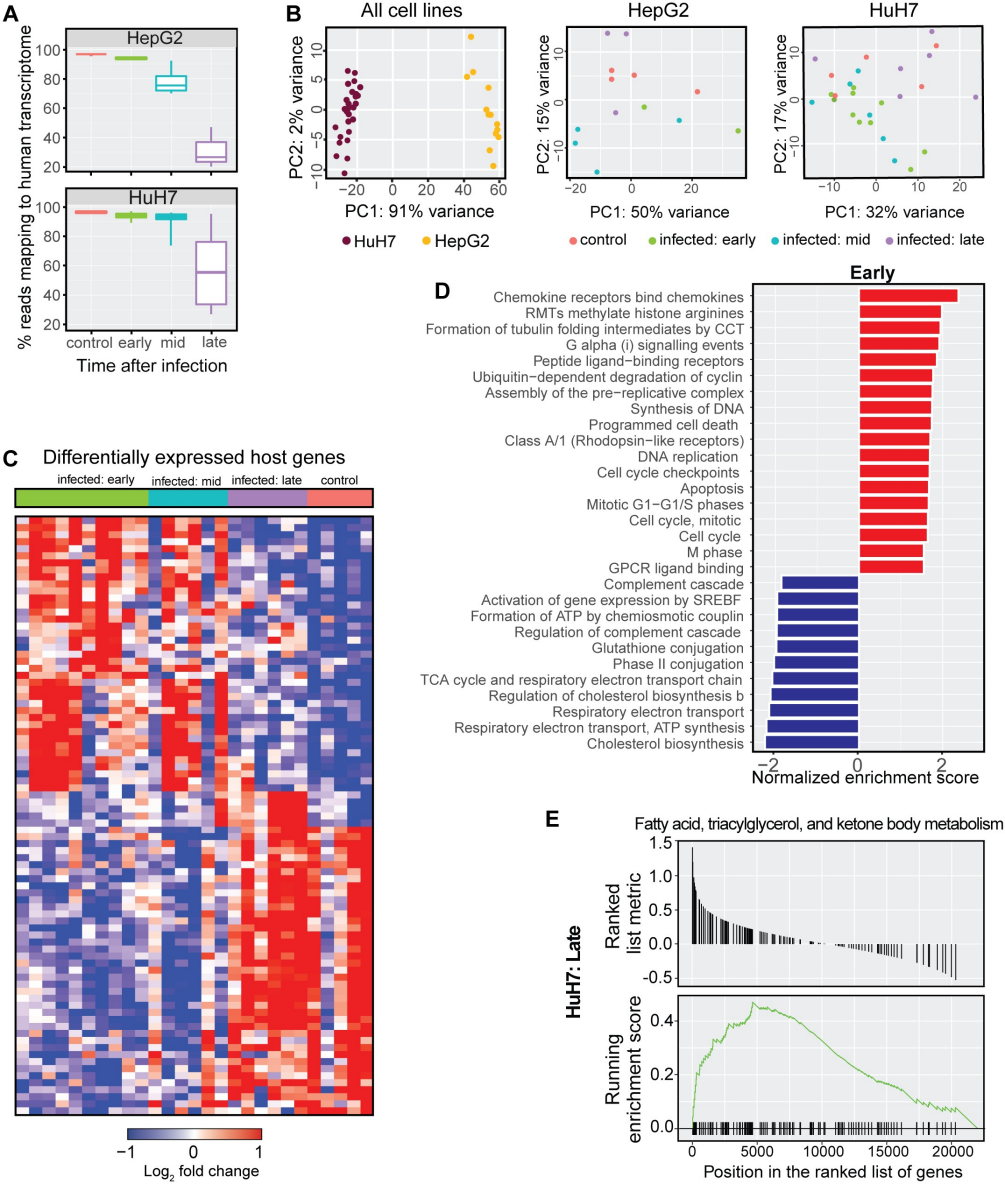
Gene expression patterns associated with *P*. *berghei* hepatocyte infection at various times post infection. (A) Percentage of reads mapping to the human transcriptome decreases with time after infection, with the unmapped reads mapping to *P*. *berghei*. (B) Principal component analysis on all samples shows grouping by cell lines accounts for 91% of variability, while PCA on the individual cell lines did not reveal discreet clusters. (C) Heatmap of differentially expressed genes (adjusted *p* < 0.05) show unique expression profiles of the for early, mid, and late time points after infection. (D) Pathways statistically enriched among up- or down-regulated genes in “early” *P*. *berghei*-infected HuH7 hepatocyte as determined by gene set enrichment analysis (GSEA). (E) GSEA plot for a representative gene set (fatty acid, triacylglycerol, and ketone body metabolism) from the “late” *P*. *berghei*-infected HuH7 hepatocytes that is statistically enriched (adjusted *p* < 0.05).

Our initial RNA-seq screen enabled the identification and validation of AQP3 as an essential host protein for *Plasmodium*, highlighting the potential of our transcriptomic approach to discover new biology. To more broadly explore host pathways that may be critical to *Plasmodium*, we performed RNA-seq on additional time points throughout *P*. *berghei* infection of HepG2 and HuH7 hepatocytes. Infected cells were collected at early (2–12 hpi), mid (18–24 hpi), and late (36–48 hpi) time points. Uninfected controls were also collected for both cell lines. In total, we performed RNA sequencing on 40 samples (S4 Table). We observed a steady decrease in the proportion of reads mapping to the human transcriptome as a function of time after infection in both HuH7 and HepG2 cells (Fig 7A). This decrease was expected due to the rising parasite occupancy of the host cell at later time points and reads that did not map to the human genome largely mapped to *P*. *berghei*. Principal component analysis (PCA) on the gene expression data showed HepG2 and HuH7 clustered separately, but such discreet clustering was not observed for individual time points within a cell line (Fig 7B).

We performed a differential gene expression analysis for the infected hepatocytes collected at early, mid, and late *P*. *berghei* infection compared to uninfected controls of the respective cell line (S5 Table). DEGs (adjusted *p* < 0.05) are shown for *P*. *berghei-*infected HuH7 cells (Fig 7C). The majority of host genes that are differentially expressed were found to be associated with the early and mid-time points. Genes involved in cell cycle (ARG1), immune processes (IL8) and the chemokine responses (CXCL family of genes) were among the most highly upregulated, while genes involved in transcriptional regulation and apoptosis (MYCN) were down-regulated. Other genes of interest to be upregulated included MTRNR2L1 and PHLDA1, two anti-apoptotic factors. We then performed a gene set enrichment analysis using GSEA [34] to identify gene sets and pathways associated with *P*. *berghei* infection at various stages of infection (S6 Table, S7 Fig). Interestingly, cholesterol biosynthesis was the most highly downregulated pathway during the early- and mid-time points following *P*. *berghei* infection (Fig 7D). Conversely, the fatty acid, triacylglycerol, and ketone body metabolism pathway was significantly upregulated during the late stage of *P*. *berghei* hepatocyte infection (Fig 7E). These data suggest that genes in the host hepatocyte are temporally regulated to first avoid clearance and prevent cell death after invasion, and then to ensure a favorable environment for growth and replication.

## Discussion

Elucidating the complex interplay between the *Plasmodium* parasite and its obligatory host liver cell is critical to understanding how a single sporozoite transforms into thousands of merozoites. Here we use the transcriptional changes that occur in HepG2 hepatocytes upon *P*. *berghei* infection to identify host factors that may be important for *Plasmodium* growth and development. We were particularly interested in genes highly induced upon infection, as the protein products of these genes could be utilized by the developing parasite. To test this possibility, we selected AQP3, one of the most significantly differentially expressed genes in infected HepG2 cells (*p* = 1.55 x 10^−7^), for further study. Through an independent secondary study with qRT-PCR analysis, we confirmed that AQP3 mRNA levels increase in *P*. *berghei*-infected HepG2 and HuH7 hepatoma lines.

Aquaporins are a family of water channel proteins that are found in most living organisms and are essential for the rapid movement of water to maintain cellular homeostasis [35]. AQP3 is an aquaglyceroporin, a unique aquaporin subtype that is highly permeable to both glycerol and water [36], but is not generally expressed in hepatocytes at the protein level (proteinatlas.org). Due to the selective induction of AQP3, but not other proteins within the aquaporin family in response to *Plasmodium* infection, we predicted a critical role during the parasite’s liver stage as a nutrient source for the developing parasite. Using immunofluorescent microscopy, we show that host AQP3 protein is more abundant in *Plasmodium* infected hepatocytes compared to uninfected cells and that the protein localizes to the PVM. The PVM, which is derived from the host cell membrane, is modified by the loss of host proteins and the integration of secreted parasite proteins [10, 37–39]; however, few host proteins are known to be trafficked to the PVM after *Plasmodium* infection of hepatocytes. Host autophagy markers, such as LC3, surround the PV of infected hepatocytes where they can integrate into the PVM as a pathogen clearance mechanism by the host cell. In roughly half of infections, LC3 integration into the PV successfully recruits lysosomes that clear the parasitic infection [40]. *Plasmodium* infections that are not successfully cleared by the host cell are able to fully mature and eventually lose the LC3 localization around the PV. Unlike the recruitment of LC3 and other autophagy markers to the PVM, here we demonstrate the incorporation of host AQP3 into the PVM in a manner that is beneficial for the growth and development of the intrahepatic parasite. We also observe the localization of AQP3 to the PVM of *P*. *falciparum*-infected erythrocytes, as previously reported [22]. These data suggest a conserved role of human AQP3 in *Plasmodium* development in multiple stage of the parasitic life cycle as well as multiple species of *Plasmodium*. Furthermore, transcriptomic studies of mice chronically infected with *T*. *gondii* show a 17-fold increase in host AQP3 mRNA expression in brain cells [41], suggesting the role of AQP3 could be conserved in other apicomplexans.

The observations that AQP3 expression is induced in response to infection and that the protein localizes to the PVM, led us to hypothesize that AQP3 is essential for *Plasmodium* development during the liver stage. The importance of AQP3 for intrahepatic *Plasmodium* development is demonstrated by the significant decrease in *P*. *berghei* parasite load upon AQP3 gene depletion (4 targeting siRNA) and genetic disruption (4 different CRISPR cell lines). Gene depletion of all other aquaporins expressed in hepatocytes indicate that AQP7 and AQP9 also contribute to *Plasmodium* development, although to a lesser extent than AQP3. AQP7 and AQP9 belong to the aquaglyceroporin family of proteins, but their expression is not induced in response to *Plasmodium* infection. In contrast, gene depletion of orthodox aquaporins did not influence parasite load. The redundant function of AQP7 and AQP9 may be responsible for the 20% parasite load observed in the AQP3^mut^ hepatocytes, where EEFs were significantly reduced in size. Taken together with our data that show AQP3 localizes to the PVM, our work suggests that AQP3 expression is co-opted by the *Plasmodium* parasite and trafficked to the PVM for survival.

We predict that AQP3 is recruited and integrated into the PVM to enable nutrient transport. *Plasmodium* is an obligate intracellular pathogen that requires the host environment for development. In particular, the rapid replication of the parasite within the PV necessitates a high demand of nutrients for parasite membrane formation and organelle synthesis. All newly synthesized membranes of the developing parasite rely on glycerol as a precursor as it is the backbone of lipid and phospholipid synthesis [42, 43]. *Plasmodium* can scavenge host lipids for the rapid assembly of membranes and organelles [44, 45]. The host lipids are tethered to glycerol and subsequently incorporated into cell membranes as phospholipids. The parasite is also able to synthesize some nutrients de novo through the FASII pathway, a fatty acid synthesis pathway that is distinct from mammalian biosynthetic pathways [46]. The FASII pathway has been found to be especially important during the liver stage of the *Plasmodium* life cycle [47] and several components of this pathway require glycerol precursors that could be obtained from the host cell. The fatty acid products of the FASII pathway are then incorporated into essential lipids, such as glycerophospholipids and acylglycerols [48, 49]. Thus, glycerol is an important component in both the de novo synthesis of fatty acids as well as the synthesis of plasma membranes from host-scavenged fatty acids.

The importance of glycerol in parasite development has been demonstrated in *P*. *falciparum* glycerol kinase knockout parasites (Δ*Pf*GK) [50]. Glycerol kinase is an enzyme that mediates the phosphorylation of glycerol to form glycerol-3-phosphate (G3P), an essential component of glycerophospholipids. Upon *Pf*GK deletion, parasite proliferation was reduced by approximately 45%, which corresponded to a 50% reduction in the incorporation of ^14^C-glycerol. This incorporation of ^14^C-glycerol from the host environment suggests unknown mechanisms of the parasite to utilize carbon sources other than glycerol kinase. Taken together, these studies show that nutrient requirements of the *Plasmodium* parasite are complex, requiring abundant resources from the host cell and that aquaporins are a protein family that may facilitate this process. The possible requirement of AQP3 for nutrient acquisition is particularly intriguing in light of a previous study that suggested the PVM is a molecular sieve for small molecule movement [51]. Using dyes of varying sizes, the selectivity filter of the PVM was proposed to be linked to molecular weight, where small molecules like glycerol are predicted to diffuse freely. Our observations suggest a more complex filtering of solutes occurs, which would enable greater control of the chemical components within the PV.

The role of aquaporins in *Plasmodium* infections has been studied for many years, but the research has primarily focused on the parasite aquaporin. *Plasmodium* encodes an aquaglyceroporin, AQP [42], which has been shown to localize to the parasite plasma membrane where it transports glycerol to the parasite [21]. Knockout of the parasite AQP significantly reduces the parasite growth rate but does not affect viability during the blood stage [21]. Based on these observations, it has been proposed that the parasite aquaporin is a potential drug target [52]. Our data suggest that human AQP3 is recruited to the PVM in hepatocytes to facilitate liver stage infection. We predict that AQP3 is important for the delivery of nutrients, possibly glycerol, through the PV, which enables the parasite AQP to then transport the nutrients into the parasitic cytoplasm to facilitate rapid growth.

Depletion of AQP3 transcripts or genetic disruption of the gene in hepatocytes did not influence liver cell viability, which was expected as the protein is not generally expressed in hepatocytes and AQP3 knockout in mice has been previously shown to be non-lethal [24]. These observations suggest that a therapeutic window for host AQP3 inhibition may be found that reduces parasite load without inhibiting the host cell. To this end, we identified the AQP3-targeting auphen [32] as a compound that reduces parasite burden during the liver (*P*. *berghei*) and asexual blood stages (*P*. *falciparum*) of the parasite’s life cycle. The efficacy of auphen in inhibiting parasite growth in both hepatocytes and erythrocytes can likely be attributed to the conserved localization of AQP3 to the PVM in both the liver and blood stages. Interestingly, AQP3 is highly abundant in human erythrocytes where it is considered the main channel for glycerol transport [27]. Our data showing liver stage *P*. *berghei* and blood stage *P*. *falciparum* inhibition of parasite load by auphen suggests that AQP3 is a host factor that two *Plasmodium* species (mouse- and human-infective) rely on in different parasite stages.

Inhibition of *P*. *falciparum* infected erythrocytes by auphen had similar phenotypic characteristics to liver stage parasite inhibition. When *P*. *falciparum* was treated with auphen for 40 hours starting at the early schizont stage, a significant reduction in parasite load was observed. We predict auphen’s inhibitory effects do not target parasite invasion, but likely prevents schizonts from properly developing mature merozoites capable of invading new erythrocytes. Though confirmation studies are necessary, this hypothesis is supported by the observation that parasites that have invaded new erythrocytes during auphen treatment exhibit delayed development into trophozoites.

Auphen is a gold-based compound that inhibits glycerol transport of human AQP3, but only minimally effects water and urea transport at high concentrations (>100 μM) [33]. It was first described for the inhibition of AQP3, but has since been shown to inhibit AQP7 glycerol transport [53]. However, it does not inhibit other orthodox aquaporins, such as AQP1 [32]. The potency of auphen against liver stage parasites was the same when the drug was administered at 0 or 24 hpi (EC_50_ ~ 0.8 μM), indicating the target was not important for invasion or early stage parasite development. Auphen has been tested in the context of cancers and shown to inhibit glycerol transport in erythrocytes with an EC_50_ = 0.80 ± 0.08 μM [32], similar to our observed *P*. *berghei* inhibition in hepatocytes by auphen. During the first 12 hours of *P*. *berghei* infection of hepatocytes, the parasite undergoes morphological changes into the EEF and by 24 hours the volume of the EEF grows four-fold before it begins to divide [8]. The parasite then divides rapidly 24–55 hpi which requires large amounts of nutrients for the synthesis of membranes and organelles. Because auphen inhibits parasite development when administered at 24 hpi, it suggests that AQP3 is needed during the late stage of *Plasmodium* hepatocyte infection. This is also supported by our results showing that parasite load is not inhibited when it is assessed at 11 hpi after treatment with auphen at the time of infection. Additionally, incubating parasites with auphen prior to infection did not prevent invasion and replication. This lack of inhibition upon parasite pre-incubation suggests that auphen does not inhibit the parasite AQP to influence invasion under our experimental conditions, as the compound is known to be an irreversible inhibitor of aquaglyceroporins active site cysteine residues [33]. Unfortunately, the complementary host preincubation study to determine auphen specificity to AQP3 is complicated by the fact that the protein is only upregulated many hours after infection. Indeed, host cell pretreatment with auphen did not influence parasite load in liver cells, suggesting that the target is translated after infection. But we cannot rule out other potential parasite or host proteins that may be a target for auphen. However, the similarities in infection phenotypes between AQP^mut^ cells and auphen treated cells support the targeting of host AQP3 by auphen.

In order to obtain a more robust understanding of the host response to *Plasmodium* infection during the liver stage, we performed RNA-seq of an additional 28 samples of an alternate hepatocyte cell line, HuH7, infected with *P*. *berghei*. Principle component analysis indicated that 91% of the variation observed within the 40 samples we collected could be attributed to different expression profiles of the two cell lines, HuH7 and HepG2. In agreement with a past microarray study that reported changes to host hepatocyte transcriptome expression in response to *Plasmodium* infection [17], we find that early time points (2–12 hours) after *P*. *berghei* infection accounted for the majority of differentially expressed genes. Some of the most highly upregulated genes included anti-apoptotic MTRNR2L1 and PHLDA1 –an exciting finding as several studies have shown that apoptosis is inhibited during *Plasmodium* invasion of hepatocytes [54, 55]. Gene set enrichment analysis also indicated that cholesterol biosynthesis genes are downregulated at early and mid-time points after infection, an interesting observation as it has been shown that *Plasmodium* relies on cholesterol acquisition from the host hepatocyte [56]. However, similar to studies that have shown an increase in cell metabolism during the blood stage [43, 57], we find that genes that are involved in metabolic pathways, such as fatty acid, triacylglycerol, and ketone body metabolism, are enriched among the genes upregulated upon *P*. *berghei* infection. The activation of this metabolic pathway during the late stage could suggest a breakdown of stored nutrients within the host liver cell that can then be utilized by the developing parasite. Future work dissecting the genes in these pathways will be critical to better understanding the host response to infection. Together our dataset of two hepatocyte cell lines, HepG2 and HuH7, is a rich resource for identifying future areas of functional research in host-pathogen interactions during the liver stage of malaria.

In this work, we have uncovered a strategy by which *Plasmodium* may secure small molecules in hepatocytes while remaining protected within the PV. Our study suggests that the parasites have a route to induce expression of host AQP3, which is incorporated in to the PVM to ensure development. Further studies investigating metabolism within the PV will be critical for testing if AQP3 is importing glycerol as a nutrient source for the parasites, as it remains possible that the parasites rely on AQP3 to maintain homeostasis or remove toxic waste. However, no strategies currently exist to separate viable parasites in the PV from the host liver cell and the low infection rate of hepatocytes hinders metabolic labeling studies. The identification of AQP3 and other potential host factors that are differentially regulated throughout the liver stage of malaria has implications for understanding host-parasite interactions. Understanding these interactions may in turn enable the development of prophylactic measures to prevent malaria. Thus, elucidating how *Plasmodium* alters host gene expression can further enhance our understanding of the host-pathogen processes that enable the parasites to cause disease. To that end, we have generated a comprehensive data set of DEGs at early, mid and late *P*. *berghei* infection of hepatocytes. We believe this data will be a valuable resource for further uncovering interactions between the *Plasmodium* parasite and its host liver cell.

## Materials and methods

### Cell and parasite culturing

HepG2 were purchased from ATCC and HuH7 cells were a kind gift from Dr. Peter Sorger (Harvard Medical School). Hepatocytes used for *P*. *berghei* infections were maintained in Dulbecco’s Modified Eagle Medium (DMEM) with L-glutamine (Gibco) supplemented with 10% heat-inactivated fetal bovine serum (HI-FBS) (v/v) (Sigma-Aldrich) and 1% antibiotic-antimycotic (Thermo Fisher Scientific) in a standard tissue culture incubator (37°C, 5% CO_2_). *Anopheles stephensi* mosquitoes infected with luciferase- or GFP-expressing *P*. *berghei* ANKA were purchased from the NYU Langone Medical Center Insectary Core Facility. Sporozoites used for liver stage experiments were harvested from freshly dissected salivary glands of mosquitoes.

*P*. *falciparum* parasites of the 3D7 strain (ATCC) were maintained in red blood cells (Golf Coast Regional Blood Center) cultured in RPMI 1640 medium supplemented with 0.5% (m/v) AlbuMAX II, 25 mM HEPES, 25 ug/mL gentamycin, 24 mM sodium bicarbonate and 50 μg/mL hypoxanthine at a pH of 7.2 and maintained at 37°C under 92% N_2_, 5% CO_2_, and 3% O_2_. Synchronization was performed using 5% sorbitol as previously described [58].

### Collection of samples for RNA-sequencing

T25 flasks were seeded with 3x10^5^ HepG2 or 8x10^4^ HuH7 cells. Cells were infected with 1x10^5^ GFP-expressing *P*. *berghei*-ANKA sporozoites 24 hours after seeding. Infected cells and uninfected controls were sorted at various times post-infection directly into lysis buffer using the BD FACSAria II cell sorter (BD Biosciences) at the Duke Human Vaccine Institute. Sytox blue was used as a live/dead cell indicator (Thermo Fisher Scientific). RNA was extracted using SMART-seq v4 Ultra Low Input RNA Kit for Sequencing (Clonetech) and libraries were prepared at the Duke Next Generation Sequencing Core Facility and sequenced on the Illumina HiSeq 4000 as 50 base pair single-end reads.

### RNA-sequencing analysis

FASTQ files comprising reads from RNA-seq were tested for quality using FastQC v.0.11.5 [59]. Adaptor sequences and low quality reads were trimmed using Trimmomatic v.0.36 [60]. Next, alignment was performed using the STAR v.2.5.1a aligner [61] to map reads to the reference *Homo sapiens* GRCh37.82 genome. PCR duplicates were marked using Picard v.2.8.2 (http://broadinstitute.github.io/picard/). The final output was a matrix of read counts per transcript. We used DESeq2 [62] to normalize the read count matrix, and to perform differential analysis. Geneset enrichment analysis was performed using GSEA [34, 63]. All statistical analyses and plots were generated using R v3.4.3.

### qRT-PCR

HepG2 or HuH7 cells infected with GFP-expressing *P*. *berghei* ANKA were sorted along with uninfected controls at 48 hpi directly into lysis buffer. RNA from samples were extracted using the Quick-RNA microprep (Zymo) according to manufacturer’s protocol. cDNA was synthesized using GoScript reverse transcriptase (Promega) according to manufacturer’s protocol. Relative abundance of *H*. *sapiens* AQP3 was quantified using a SYBR Green Light Cycler 480 (Roche) according to manufacturer’s protocol in a 96-well plate. Relative abundance of AQP3 expression in *P*. *berghei*-infected cells was compared to uninfected cells and normalized to the *H*. *sapiens* 18S housekeeping gene (primers listed in S1 Table). qRT-PCR was run in technical triplicates. RNA was isolated for 3 biological replicates for HepG2 cells and 4 biological replicates for HuH7 cells.

### Immunofluorescence microscopy

HepG2 and HuH7 cells (2.5x10^5^) were seeded on cover slips in 24-wells plates and infected with *P*. *berghei* with a multiplicity of infection (MOI) of 1:4. Cells were fixed at various times post infection in 4% paraformaldehyde for 20 minutes at room temperature. Cells were washed three times in PBS and were subsequently blocked with 3% BSA for 45 minutes at room temperature. Cells were incubated in primary antibodies, rabbit-anti *Pb* heat-shock protein 70 [64], rabbit anti-*Hs*AQP3 (Rockland), and goat anti-UIS4 (LSBio) that were diluted 1:100, 1:300, and 1:1,000, respectively, for 1.5 hours at room temperature or 4°C overnight. Cells were washed three times with PBS and incubated in secondary antibodies, AlexaFluor 488 goat anti-mouse and AlexaFluor 568 donkey anti-rabbit diluted 1:400 (Thermo Fisher Scientific), for 1 hour at room temperature. For UIS4, staining AlexaFluor 488 donkey anti-goat was used at 1:5,000. Cells were washed with PBS and incubated with 0.5 μg/mL DAPI for 7 minutes. Cells were washed once more with PBS and slides were mounted with ProLong Gold Antifade (Thermo Fisher Scientific). iRBCs samples were fixed, permeabilized and blocked as described previously [65]. Incubations with anti-*Hs*AQP3 primary antibody and secondary antibody were completed as described for *P*. *berghei*. After nuclear staining in 1 μg/mL Hoechst 33342, cells were imaged on a widefield fluorescent microscope.

Images were taken using the Zeiss Axio Observer wide field fluorescence microscope or the Zeiss 880 Airyscan inverted confocal. 3D stacks were acquired on a Zeiss LSM 510 inverted confocal microscope with a 100x 1.4 NA Plan-Apochromat oil objective. 3D images were constructed and colocalization parameters were determined using Imaris software (Bitplane). A minimum of three independent experiments were completed for all localization studies. EEF size quantification was completed using ImageJ. Number of EEFs for AQP3^mut^ cells and auphen treated cells were quantified/well in 384-well plates. Three independent experiments were performed with 5–10 wells/condition in each experiment.

### *Plasmodium* inhibition assays

Parasites isolated from salivary glands of infected *A*. *stephensi* were used to infect HuH7 and HepG2 hepatocytes as previously described [66]. Cell viability and parasite load were assessed using CellTiter-Fluor (Promega) and Bright-Glo (Promega) reagents, respectively, according to manufacturer’s protocols. Fluorescence and luminescence readouts were collected using the EnVision plate reader (Perkin Elmer). For inhibition assays, auphen was synthesized [67] and 20–0.01 μM of compound was added to *P*. *berghei* ANKA-infected HuH7/HepG2 cells or *P*. *falciparum* Dd2-infected erythrocytes 10 minutes post infection in a dose-dependent manner with DMSO normalized to 1% in all wells. *P*. *berghei* parasite load upon auphen treatment was quantified using the luciferase reporter as described above in biological triplicate. *P*. *falciparum* Dd2 parasite load was assessed with DAPI nuclear staining as previously described in biological duplicate [66].

For auphen pre-incubation studies, freshly harvested sporozoites from mosquito salivary glands were incubated with 2–10 μM auphen for 30 minutes at room temperature. As a control, sporozoites were also incubated with DMSO and all treatments had 1% DMSO. After incubation, the compound was removed by centrifugation and resuspension of sporozoites in untreated media. Cells were pretreatment with the same concentrations of auphen. After a 30 minute incubation with auphen or DMSO control (1%), media was gently aspirated and fresh media was added to the wells.

For auphen treatment of *P*. *falciparum*, synchronized iRBCs in the schizont stage were treated with 2 μM auphen for 40 hours. After 40 hours, Geimsa (Sigma-Aldrich) stained blood smears were imaged on a brightfield microscope. Parasitemia was calculated by counting the number of *P*. *falciparum* infected red blood cells out of a total of 1,200 cells.

### Depletion of aquaporin expression by RNA interference

HuH7 cells were reverse transfected with 50 nM siRNAs (S1 Table) at 2x10^3^ cells/well in 384-well plates. siRNAs (Qiagen) were transfected using Lipofectamine 2000 (Thermo Fisher Scientific) according to manufacturer’s protocol. Cells were reverse transfected in 6 technical replicates and in duplicate plates. One plate was used to assess RNA knockdown efficiency 48 hours post transfection using Quick-RNA MicroPrep (Zymo). cDNA was generated using GoScript (Promega) and qRT-PCR was performed as previously described to assess the knockdown of genes of interest compared to mock-transfected cells. Primers that were used for the qRT-PCR are listed in S1 Table. 48 hours post transfection the duplicate plate was infected with 4x10^3^ luciferase-expressing *P*. *berghei* ANKA sporozoites per well. 48 hpi cell viability and parasite load were assessed as described above. Each experiment was completed with 6 technical replicates and three independent experiments were performed.

### Generation of AQP3 mutant cell line

Four separate AQP3^mut^ cell lines were generated using different guide RNAs (gRNA). The gRNA sequences were determined (crispr.mit.edu) for recruitment of Cas9 to either the first or second exon of AQP3 and are listed in S3 Table. Tails were added to gRNA sequence for introduction into px330^34^ (Addgene plasmid #42230), a plasmid containing a human codon-optimized SpCas9. px330 was digested using BbsI (NEB) for one hour according to manufacturer’s protocol and gel purified. Reverse complements of gRNA were annealed using T4 ligation buffer (NEB). Linearized px330 and gRNA were ligated using T4 ligase (NEB) according to manufacturer’s protocol. Ligated plasmid was transformed into XL-Gold competent cells and plasmids were isolated using a standard mini-prep kit (Qiagen). Sequencing was verified using Eton BioScience Inc.

HuH7 cells (2x10^5^/per well) were seeded in a 6-well plate in DMEM. Cells were co-transfected with pX330, containing the gRNA sequence, and pCDNA3.0 containing a blasticidin resistance marker using Lipofectamine 2000 (Thermo Fisher Scientific) according to manufacturer’s instructions. Cells that were transfected with gRNAs targeting exon 2 were transfected with two separate gRNA-containing px330 plasmids, homologous to the second exon of AQP3 and 6 nucleotides away from one another. Mock cells containing no plasmids were included. HuH7 cells containing plasmids were then trypsinized and replated into 10 cm tissue culture plates in cDMEM 24 hours after transfection. Cells were washed and then cDMEM+5 μg/mL blasticidin was added 48 hours after transfection. Mock cells were monitored for cell death daily until no surviving cells remained. Transfected HuH7 cells were then single-cell sorted into 96-well plates and maintained in standard culture medium as described above. Populations were grown from single cells and expression of AQP3 and Actin RNA was assessed by RT-PCR (AQP3 F: ATGGGTCGACAGAAGGAGCT, R:TCAGATCTGCTCCTTGTGCTT; ACTB F: GCCTCGCCTTTGCCGA, R: GTTGAAGGTCTCAAACATGATCTGG). RNA was extracted and cDNA synthesized as previously described and genomic DNA (gDNA) was purified using the Quick-DNA microprep (Zymo). Primers to amplify both exons of the AQP3 gene were designed as well as for the full-length AQP3 mRNA (S1 Table). Clonal populations showing no expression of the full-length transcript were selected for further evaluation and designated as AQP3^mut^ cell lines. Parasite load in the AQP3^mut^ cell lines infected with luciferase- or GFP-expressing *P*. *berghei* were compared to wildtype HuH7 cells as previously described.

### Auphen synthesis

Reactions were carried out under a nitrogen atmosphere with dry solvents and oven-dried glassware under anhydrous conditions unless specified otherwise. Reagents were purchased at the highest commercial quality and used without further purification, unless otherwise stated. Yields refer to chromatographically and spectroscopically (^1^H and ^13^C NMR) homogeneous materials, unless otherwise stated. NMR spectra were recorded on a 400 MHz Varian Inova spectrometer instrument, and were calibrated using residual undeuterated solvents as internal reference (dimethylsulfoxide, δ = 2.50 ppm, ^1^H NMR; 39.52 ppm, ^13^C NMR). Chemical shifts (δ) are reported in parts per million (ppm); NMR peak multiplicities are denoted by the following abbreviations: s = singlet, d = doublet, t = triplet, q = quartet, p = pentet, dd = doublet of doublets, dt = doublet of triplets, m = multiplet, br = broad; coupling constants (*J*) are reported in Hertz (Hz).

[AuCl2(phen)]Cl (Auphen) was prepared as previously described [67]. Briefly, to a solution of HAuCl_4_**●**3H_2_O (100 mg, 0.254 mmol) in EtOH (1.0 mL) a solution of 1,10 phenanthroline (151 mg, 0.838 mmol) in EtOH (1.0 mL) was added slowly. The reaction was stirred at reflux for 4 hours. The reaction was cooled to room temperature, filtered and washed with cold EtOH (3 x 5 mL), affording pure auphen as orange crystals (116 mg, 0.240 mmol, 95%). ^1^H NMR (400 MHz, DMSO-*d*_6_): δ 9.31 (dd, *J* = 4.9, 1.6 Hz, 2H), 9.08 (dd, *J* = 8.2, 1.5 Hz, 2H), 8.37 (s, 2H), 8.23 (dd, *J* = 8.2, 4.9 Hz, 2H) ppm; ^13^C NMR (100 MHz, DMSO-*d*_6_): δ 147.60, 141.94, 137.37, 129.55, 127.53, 125.73 ppm.

## Supporting information

S1 Fig Gating strategy for GFP-expressing *P*. *berghei* infected hepatocytes. (A) Uninfected HepG2 cells and cells sorted (B) 4, (C) 24, and (D) 48 hours post *P*. *berghei* infection. Sytox Blue was used as a live/dead cell indicator.
(TIF)

S2 Fig Immunofluorescent imaging analysis of AQP3 signal. HuH7 cells infected with *P*. *berghei* were stained for AQP3 (red) and DAPI (white). Nuclei of *P*. *berghei* were pseudo-colored blue. Scale bar 10 μm. AQP3 staining is only found in *P*. *berghei* infected hepatocytes and localizes exclusively to the PVM.
(TIF)

S3 Fig siRNA depletion of aquaporin transcripts. (A) Cell viability of HuH7 cells treated with multiple (2) siRNAs targeting AQP3 and SR-BI and infected with *P*. *berghei*. Non-targeting scrambled siRNAs were used as a negative control. Transfection of hepatocytes with siRNAs did not affect cell viability. (B) Parasite load and (C) cell viability of HuH7 cells reverse transfected with individual siRNAs and infected with *P*. *berghei* 48 hours post transfection. Parasite load and cell viability was assessed at 48 hpi. (One-Way ANOVA, Dunnett’s multiple comparison; n = 3 individual biological experiments). Error bars represent SEM. *P < 0.05, **P < 0.01, ***P < 0.001, ****P < 0.0001.
(TIF)

S4 Fig Generation of mutant AQP3 cell lines. (A) Parasite load measured in wildtype HuH7 cells and AQP3^mut1-4^ cell lines 48 hpi. All mutant cell lines had significant reduction in parasite load, averaging 80% reduction (One-Way ANOVA, Dunnett’s multiple comparison; n = 3 independent experiments). ****P < 0.0001. (B) Amplification of AQP3 mRNA from cDNA generated from RNA extracted from wildtype cells and AQP3^mut1-4^ cell lines. AQP3^mut1^ had a 39 base pair shift in mRNA and AQP3^mut1-4^ cell lines had no detectable AQP3 mRNA. (C) Sequencing of AQP3^mut1^ genomic DNA confirming a 39 bp deletion in exon 2 of AQP3. (D) Predicted protein structure for AQP3^mut1^ compared to wildtype extrapolated using the Swiss model homology analysis. (E) Cell viability of AQP3^mut1^ compared to wildtype HuH7 cells shows no significant difference (*p* = 0.9396, unpaired Student’s *t-*test; n = 3). Error bars represent SEM.
(TIF)

S5 Fig Synthesis of auphen. (A) ^1^H NMR of [AuCl_2_(phen)]Cl (Auphen) in DMSO-d_6_ (400 MHz). (B) ^13^C NMR of [AuCl_2_(phen)]Cl (Auphen) in DMSO-d_6_ (100 MHz).
(TIF)

S6 Fig Auphen inhibits parasite load in multiple hepatoma cell lines and is effective at inhibition of *P*. *berghei* parasite load up to 24 hpi. (A) Parasite load of HepG2 cells infected with luciferase-expressing *P*. *berghei* and treated with 0.05–20 μM auphen at time of infection (*circles*) or 24 hpi (*squares*). EC_50_ = 0.62 ± 0.09 when auphen is added at 0 hpi and EC_50_ = 0.62 ± 0.12 when auphen is added 24 hpi. Luminescence is measured 48 hpi (n = 1, 4 technical replicates). (B) Cell viability measured by CellTiter-Fluor (Promega) of HuH7 cells infected with *P*. *berghei* and treated with 0.05–20 μM of auphen at time of infection. Percent cell viability is compared to DMSO treated HuH7 cells. Auphen did not lead to any significant changes in cell viability (*p* = 0.165, One-Way ANOVA; n = 3 independent experiments). (C) HuH7 cells infected with *P*. *berghei* and treated with auphen in a dose-dependent manner at time of infection. Parasite load measured by luminescence at 11 (*light blue*), 24 (*dark blue*) and 44 hpi (*black*) is plotted as log(relative luminescent units). Parasite load is normalized to cells infected with *P*. *berghei* and treated with DMSO. No inhibition of parasite is seen when measured at 11 hpi and only at the highest concentrations of auphen is there some inhibition in parasite load when measured 24 hpi. Three independent experiments were completed and showing data from a representative biological replicate. Error bars represent SD. (D) Parasite load of *P*. *berghei* infected HuH7 cells treated with auphen in a dose-dependent manner. (*Black)* Cells were treated with auphen immediately after infection and parasite load was inhibited in a dose-dependent manner. (*Light blue)* Cells were treated for 30 with auphen in a dose-dependent manner. Cells were washed with fresh media before *P*. *berghei* infection. No significant inhibition of parasite load was observed (n = 1, 3 technical replicates). Error bars represent SD.
(TIF)

S7 Fig HuH7 gene set enrichment analysis. Gene sets that have been found to be statistically significant for (A) early, (B) mid, and (C) late *P*. *berghei*-infected HuH7 hepatocytes.
(TIF)

S1 Table siRNAs used for depletion of aquaporin genes expressed in hepatocytes and primers used for qRT-PCR. Product names are for Dharmacon (scramble and SR-B1) or Qiagen (all AQP siRNAs).
(PDF)

S2 Table Parasite load, cell viability, and relative mRNA expression of targeted genes in HuH7 cells treated with individual siRNAs and infected with *P*. *berghei*. (PDF)

S3 Table List of gRNAs used for generating AQP3mut cell lineusing the CRISPR/Cas9 genome editing. (PDF)

S4 Table RNA-seq summary data. (PDF)

S5 Table Differential expression of hepatocytes infected with P. berghei. (XLSX)

S6 Table Gene set enrichment using GSEA for each cell and time point. (XLSX)

S1 Movie 3D confocal image of *P*. *berghei* infected hepatocyte. (MP4)
