# Supplementary figures and images for: Plasmodium parasite exploits host aquaporin-3 during liver stage malaria infection
Source: PLoS Pathog. 2018 May 18;14(5):e1007057. doi: 10.1371/journal.ppat.1007057 (PMC5979039; doi:10.1371/journal.ppat.1007057)

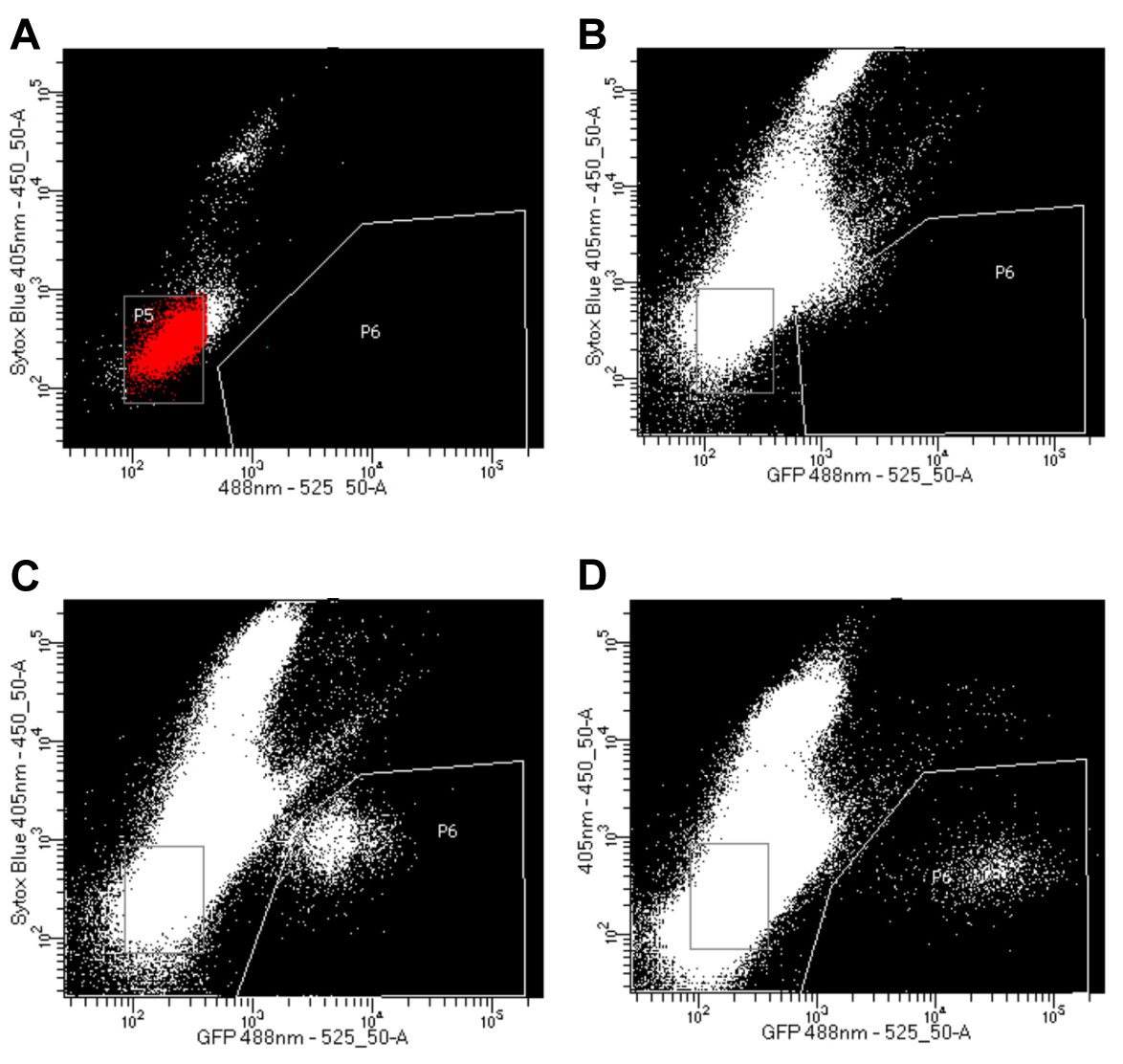

Supplement: S1 Fig — (A) Uninfected HepG2 cells and cells sorted (B) 4, (C) 24, and (D) 48 hours post P. berghei infection. Sytox Blue was used as a live/dead cell indicator. (TIF) [file ppat.1007057.s001.tif]

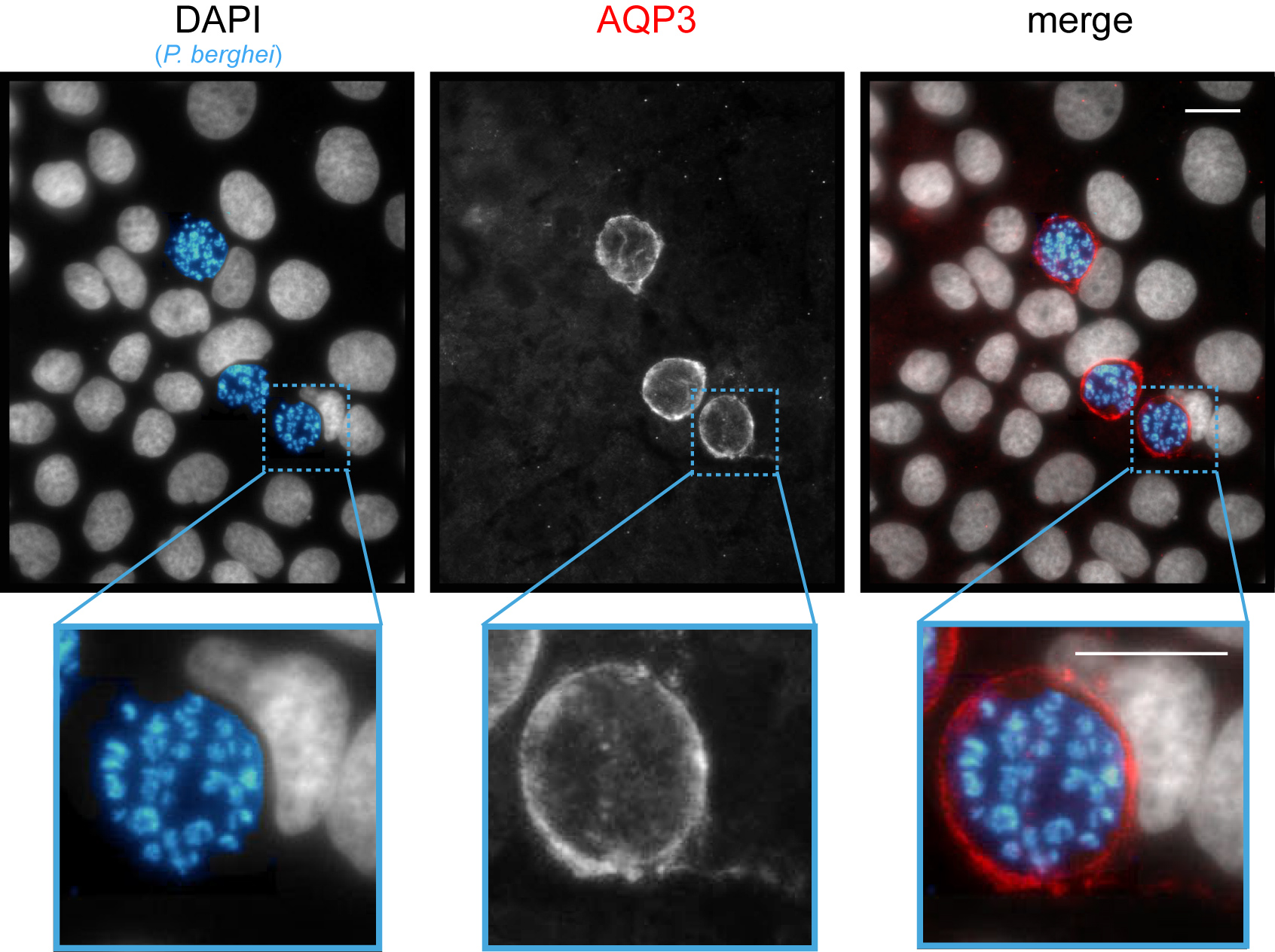

Supplement: S2 Fig — HuH7 cells infected with P. berghei were stained for AQP3 (red) and DAPI (white). Nuclei of P. berghei were pseudo-colored blue. Scale bar 10 μm. AQP3 staining is only found in P. berghei infected hepatocytes and localizes exclusively to the PVM. (TIF) [file ppat.1007057.s002.tif]

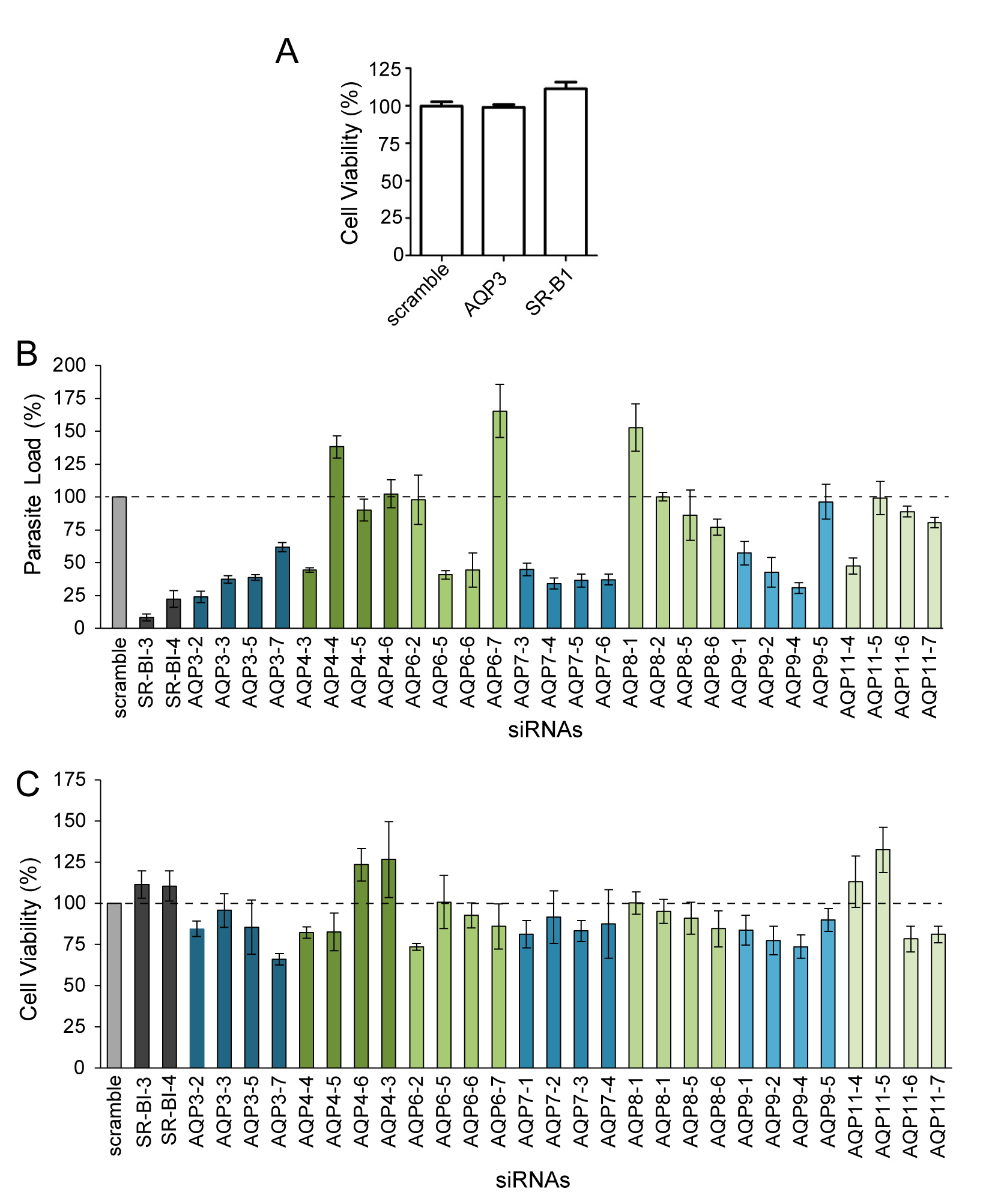

Supplement: S3 Fig — (A) Cell viability of HuH7 cells treated with multiple (2) siRNAs targeting AQP3 and SR-BI and infected with P. berghei. Non-targeting scrambled siRNAs were used as a negative control. Transfection of hepatocytes with siRNAs did not affect cell viability. (B) Parasite load and (C) cell viability of HuH7 cells reverse transfected with individual siRNAs and infected with P. berghei 48 hours post transfection. Parasite load and cell viability was assessed at 48 hpi. (One-Way ANOVA, Dunnett’s multiple comparison; n = 3 individual biological experiments). Error bars represent SEM. *P < 0.05, **P < 0.01, ***P < 0.001, ****P < 0.0001. (TIF) [file ppat.1007057.s003.tif]

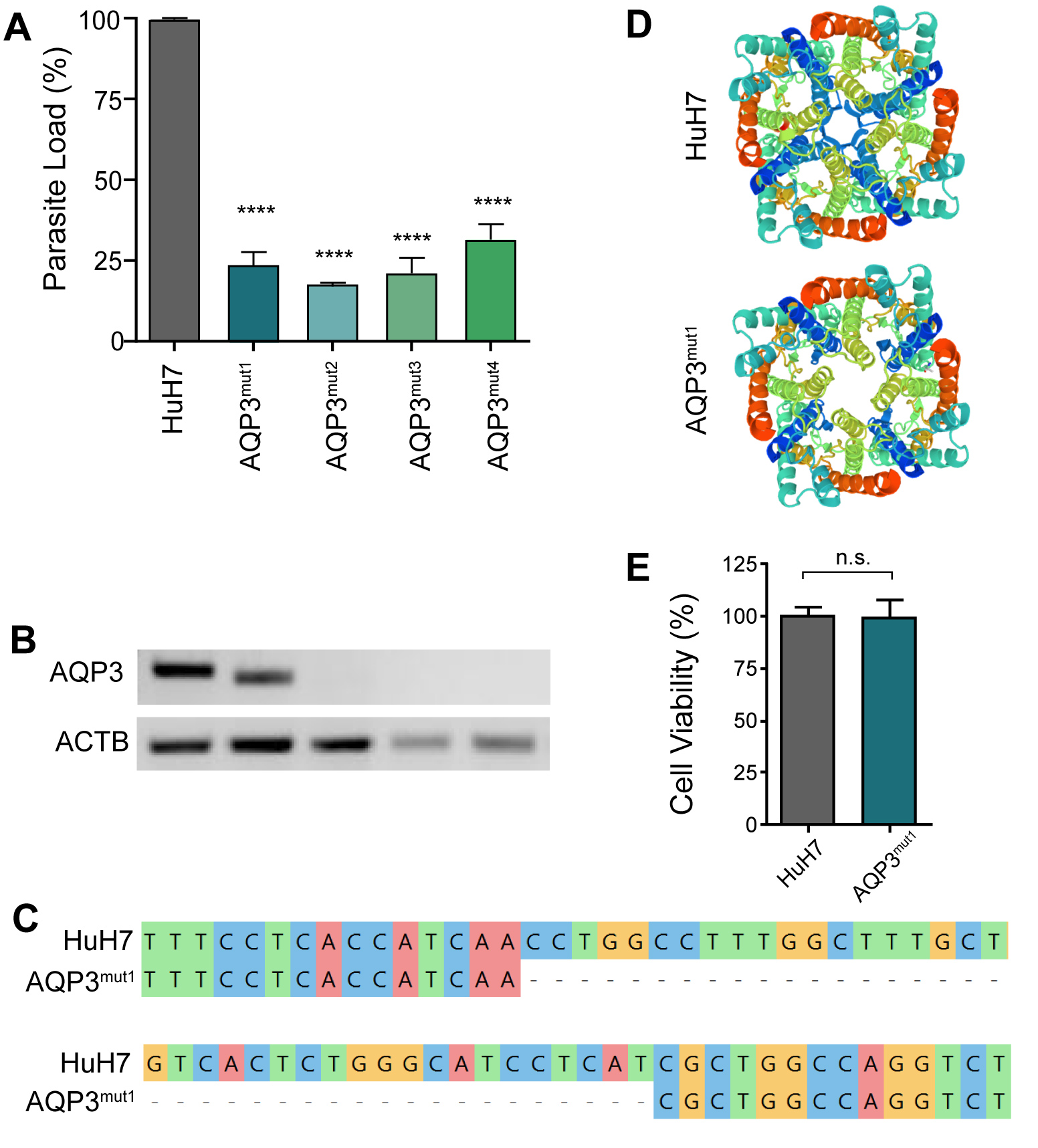

Supplement: S4 Fig — (A) Parasite load measured in wildtype HuH7 cells and AQP3mut1-4 cell lines 48 hpi. All mutant cell lines had significant reduction in parasite load, averaging 80% reduction (One-Way ANOVA, Dunnett’s multiple comparison; n = 3 independent experiments). ****P < 0.0001. (B) Amplification of AQP3 mRNA from cDNA generated from RNA extracted from wildtype cells and AQP3mut1-4 cell lines. AQP3mut1 had a 39 base pair shift in mRNA and AQP3mut1-4 cell lines had no detectable AQP3 mRNA. (C) Sequencing of AQP3mut1 genomic DNA confirming a 39 bp deletion in exon 2 of AQP3. (D) Predicted protein structure for AQP3mut1 compared to wildtype extrapolated using the Swiss model homology analysis. (E) Cell viability of AQP3mut1 compared to wildtype HuH7 cells shows no significant difference (p = 0.9396, unpaired Student’s t-test; n = 3). Error bars represent SEM. (TIF) [file ppat.1007057.s004.tif]

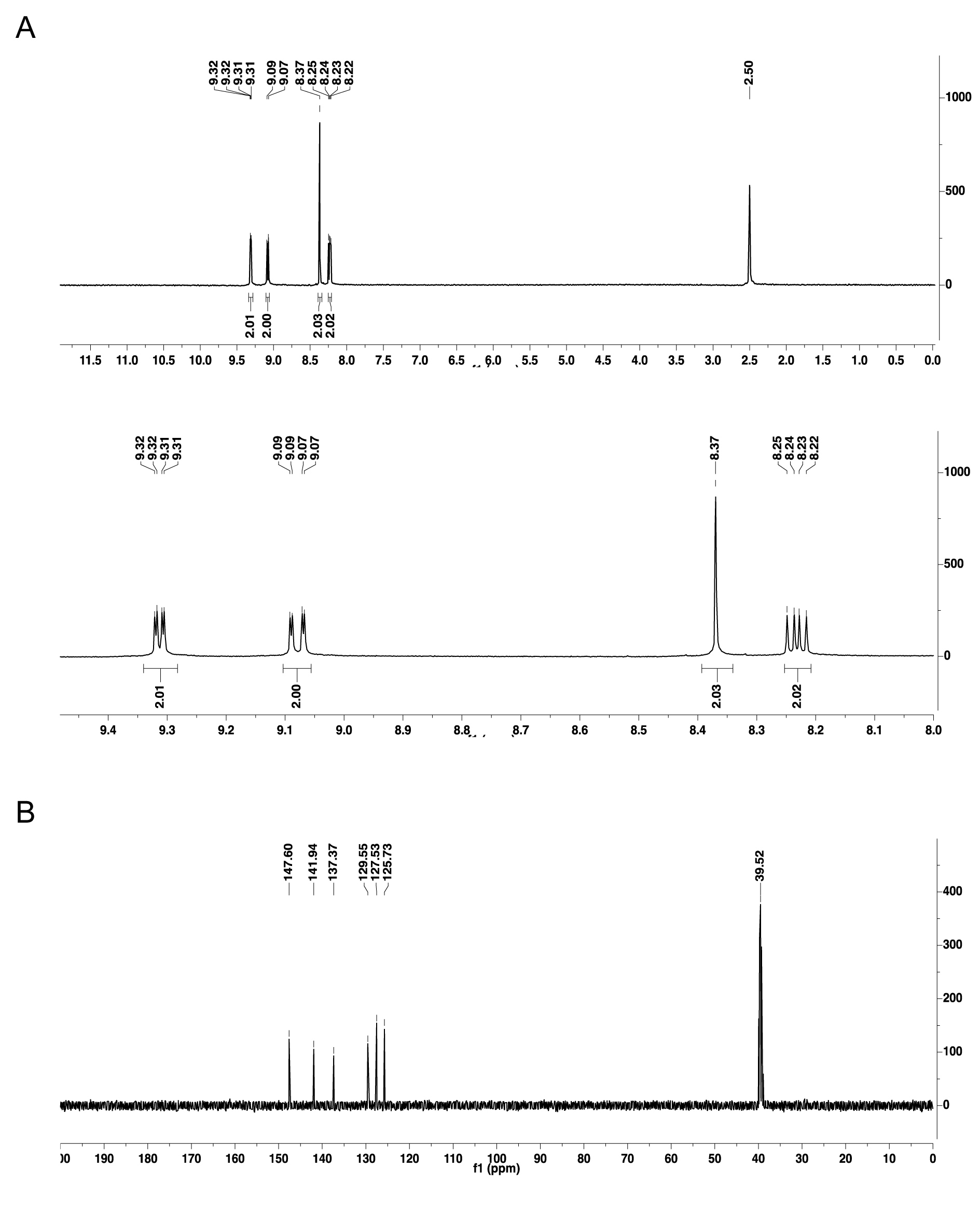

Supplement: S5 Fig — (A) 1H NMR of [AuCl2(phen)]Cl (Auphen) in DMSO-d6 (400 MHz). (B) 13C NMR of [AuCl2(phen)]Cl (Auphen) in DMSO-d6 (100 MHz). (TIF) [file ppat.1007057.s005.tif]

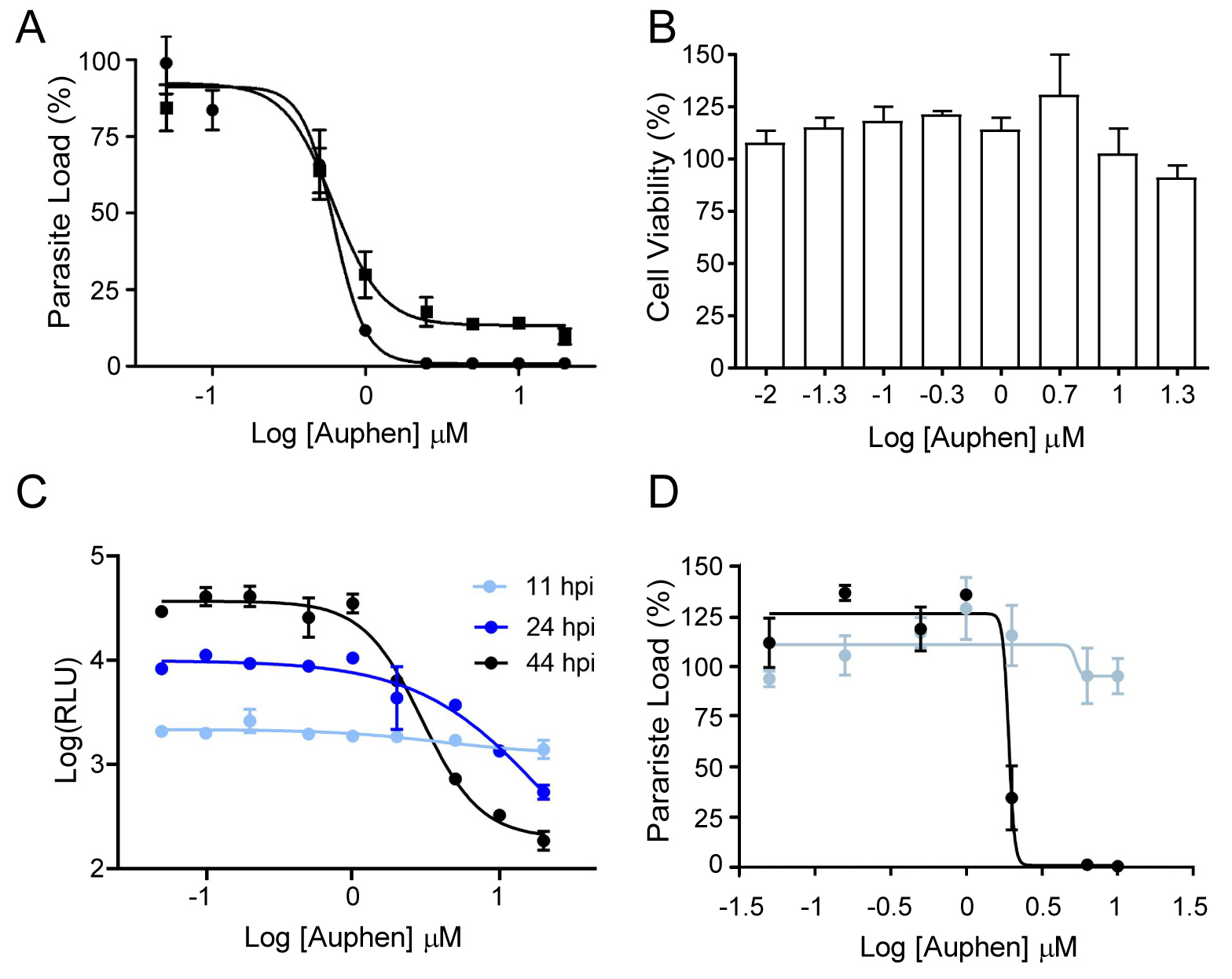

Supplement: S6 Fig — (A) Parasite load of HepG2 cells infected with luciferase-expressing P. berghei and treated with 0.05–20 μM auphen at time of infection (circles) or 24 hpi (squares). EC50 = 0.62 ± 0.09 when auphen is added at 0 hpi and EC50 = 0.62 ± 0.12 when auphen is added 24 hpi. Luminescence is measured 48 hpi (n = 1, 4 technical replicates). (B) Cell viability measured by CellTiter-Fluor (Promega) of HuH7 cells infected with P. berghei and treated with 0.05–20 μM of auphen at time of infection. Percent cell viability is compared to DMSO treated HuH7 cells. Auphen did not lead to any significant changes in cell viability (p = 0.165, One-Way ANOVA; n = 3 independent experiments). (C) HuH7 cells infected with P. berghei and treated with auphen in a dose-dependent manner at time of infection. Parasite load measured by luminescence at 11 (light blue), 24 (dark blue) and 44 hpi (black) is plotted as log(relative luminescent units). Parasite load is normalized to cells infected with P. berghei and treated with DMSO. No inhibition of parasite is seen when measured at 11 hpi and only at the highest concentrations of auphen is there some inhibition in parasite load when measured 24 hpi. Three independent experiments were completed and showing data from a representative biological replicate. Error bars represent SD. (D) Parasite load of P. berghei infected HuH7 cells treated with auphen in a dose-dependent manner. (Black) Cells were treated with auphen immediately after infection and parasite load was inhibited in a dose-dependent manner. (Light blue) Cells were treated for 30 with auphen in a dose-dependent manner. Cells were washed with fresh media before P. berghei infection. No significant inhibition of parasite load was observed (n = 1, 3 technical replicates). Error bars represent SD. (TIF) [file ppat.1007057.s006.tif]

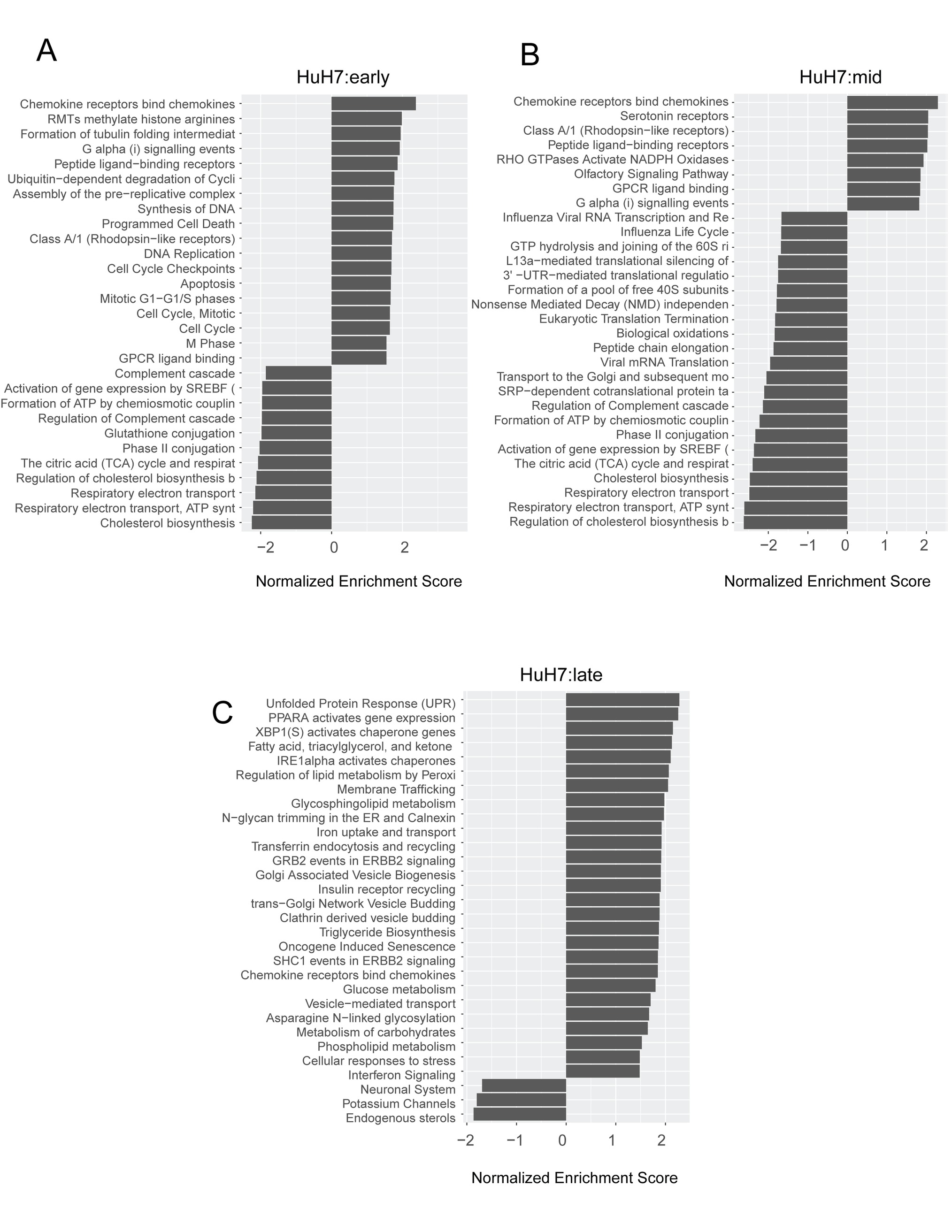

Supplement: S7 Fig — Gene sets that have been found to be statistically significant for (A) early, (B) mid, and (C) late P. berghei-infected HuH7 hepatocytes. (TIF) [file ppat.1007057.s007.tif]
