# Supplementary material for: Plasmodium parasite exploits host aquaporin-3 during liver stage malaria infection
Source: PLoS Pathog. 2018 May 18;14(5):e1007057. doi: 10.1371/journal.ppat.1007057 (PMC5979039; doi:10.1371/journal.ppat.1007057)
Supplement: S1 Table — Product names are for Dharmacon (scramble and SR-B1) or Qiagen (all AQP siRNAs). (PDF) [file ppat.1007057.s008.pdf]

S1 Table. siRNAs used for depletion of aquaporin genes expressed in hepatocytes and primers used for qRT-PCR. Product names are for Dharmacon (scramble and SR-B1) or Qiagen (all AQP siRNAs).

| NCBI gene | mRNA Accessions | siRNA Target Sequence | Product Name |
|---|---|---|---|
| scramble | non-targetting | TACATAACCGGACATAATC | D-001210-04-05 |
| SR-B1 | NM_005505 | ACTGACCGGACGTGGGATT | D-010592-03* |
| SR-B1 | NM_005505 | CCTGTTTGACCCTTCTAAG | D-010592-04* |
| AQP3 | NM_004925 | CTGGATGTACATACATAAGTA | Hs_AQP3_2 * |
| AQP3 | NM_004925 | CTGGATCAAGCTGCCCATCTA | Hs_AQP3_3 * |
| AQP3 | NM_004925 | ACCGGAATTTGGGTCAATACA | Hs_AQP3_5 |
| AQP3 | NM_004925 | AGCCGGCATCTTTGCTACCTA | Hs_AQP3_7 |
| AQP4 | NM_001650 NM_004028 | CTGGTTGAGTTGATAATCACA | Hs_AQP4_3 |
| AQP4 | NM_001650 NM_004028 | GACGGATGACCTGATTCTAAA | Hs_AQP4_4 |
| AQP4 | NM_001650 NM_004028 | ACCGGTCGACATGGTTCTCAT | Hs_AQP4_5 |
| AQP4 | NM_001650 NM_004028 | CAGGAAGATCAGCATCGCCAA | Hs_AQP4_6 |
| AQP6 | NM_001652 NM_053286 | CAGGGACTGCTGTGAAATCAA | Hs_AQP6_2 |
| AQP6 | NM_001652 NM_053286 | TTCAGTGCCTCTGAAAGACTA | Hs_AQP6_5 |
| AQP6 | NM_001652 NM_053286 | CCTGGCCTCACTGATCTACAA | Hs_AQP6_6 |
| AQP6 | NM_001652 | TGGGATCAACGTGGTCCGGAA | Hs_AQP6_7 |
| AQP7 | NM_001170 | CTGGAGGAAGTTTCCGGTCTA | Hs_AQP7_3 |
| AQP7 | NM_001170 | CGGGATAACCGTATTGCCCAA | Hs_AQP7_4 |
| AQP7 | NM_001170 | CACATATGTCATGATGGTATT | Hs_AQP7_5 |
| AQP7 | NM_001170 | CGCAGCTGTGACCTTTGCTAA | Hs_AQP7_6 |
| AQP8 | NM_001169 | CTGACAATTCTCACTTTGCAA | Hs_AQP8_1 |
| AQP8 | NM_001169 | GAGCGTGTTTCTTGAGAGGAA | Hs_AQP8_2 |
| AQP8 | NM_001169 | ATCCTGATGTCTGGAGAGATA | Hs_AQP8_5 |
| AQP8 | NM_001169 | TCCGATGTTTGTGCCATCTGA | Hs_AQP8_6 |
| AQP9 | NM_020980 | CAGCCTCTAATTGGAACGGCA | Hs_AQP9_1 |
| AQP9 | NM_020980 | ACGACTGTGCTTGTCCATTAT | Hs_AQP9_2 |
| AQP9 | NM_020980 | AACCGTCTTTGGCATTTACTA | Hs_AQP9_4 |
| AQP9 | NM_020980 | CACGTTCATCTTGATTGTCCT | Hs_AQP9_5 |
| AQP11 | NM_173039 | CTGGCTCCTTCTTTAGGTATA | Hs_AQP11_4 |
| AQP11 | NM_173039 | CTGCATAACAACCATACAATT | Hs_AQP11_5 |
| AQP11 | NM_173039 | CGAGAGGAGCTTCGCTTGCAA | Hs_AQP11_6 |
| AQP11 | NM_173039 | CTGGACGCTGACGCTCGTCTA | Hs_AQP11_7 |

| Gene | Primer Sequence |
|---|---|
| SR-BI_F | TCTCTCCGTCTACTTCTTTGACG |
| SR-BI_R | AACTGGAAGGTGCGGTACTC |
| AQP3_F | ACCAGCTTTTTGTTTCGGGC |
| AQP3_R | ACACGATAAGGGAGGCTGTG |
| AQP4_F | GCCATCATTGGAGCAGGAATCC |
| AQP4_R | ACTCAACCAGGAGACCATGACC |
| AQP6_F | CTGCTTCTGACCCTGCAGC |
| AQP6_R | GGCCCCACCCAGAAGACC |
| AQP8 F | GGGCAGAGCCCATAGTGTGA |
| AQP8 R | AGGGTTGAAGTGTCCACCAC |
| AQP7_F | GAGGAAGATGGTGCGAGAGTTC |
| AQP7_R | GAGGAAGATGGTGCGAGAGTTC |
| AQP9_F | TCTCTGAGTTCTTGGGCACG |
| AQP9_R | GGTTGATGTGACCACCAGAG |
| AQP11_F | CACTTCCAGGAAGTCCGAACCA |
| AQP11_R | GTAGCGAAAGTGCCAAAGCTGG |

*indicates siRNAs used for pooled siRNA gene depletion in Fig 3A.
