# Supplementary material for: Plasmodium parasite exploits host aquaporin-3 during liver stage malaria infection
Source: PLoS Pathog. 2018 May 18;14(5):e1007057. doi: 10.1371/journal.ppat.1007057 (PMC5979039; doi:10.1371/journal.ppat.1007057)
Supplement: S2 Table — (PDF) [file ppat.1007057.s009.pdf]

S2 Table. Parasite load, cell viability, and relative mRNA expression of targeted genes in HuH7 cells treated with individual siRNAs and infected with *P. berghei.*

	Parasite Load (%)			Cell Viability (%)			Relative mRNA
	n = 3-4			n = 3-4			n=1
Qiagen siRNA	(Mean +/- SEM)	Summary	*P* value	(Mean +/- SEM)	Summary	*P* value	%
scramble	100 ± 0.0			100 ± 0.0			100.00
SR-BI-3 †	8.59 ± 3.00	****	0.0001	111.9 ± 9.75	ns	0.999	3.45
SR-BI-4 †	22.52 ± 6.48	****	0.0001	110.52 ± 10.68	ns	0.9991	3.08
AQP3-2 †	24.03 ± 6.046	***	0.0002	84.60 ± 5.31	ns	0.9946	5.69
AQP3-3 †	37.37 ± 3.84	**	0.0046	95.72 ± 11.72	ns	0.9997	6.65
AQP3-5	38.93 ± 3.11	**	0.0063	85.64 ± 20.15	ns	0.9988	14.06
AQP3-7	61.88 ± 5.17	ns	0.273	66.10 ± 4.11	ns	0.5947	10.37
AQP4-3	44.41 ± 2.30	*	0.0187	82.35 ± 4.34	ns	0.994	22.38
AQP4-4 †	138.13 ± 12.14	ns	0.2728	82.69 ± 13.95	ns	0.9942	4.92
AQP4-5 †	90.12 ± 11.91	ns	0.9991	123.51 ± 11.97	ns	0.9565	8.48
AQP4-6	102.48 ± 14.97	ns	0.9998	126.62 ± 28.24	ns	0.8832	9.31
AQP6-2 †	97.91 ± 26.41	ns	0.9999	73.72 ± 2.51	ns	0.8239	6.47
AQP6-5	40.80 ± 4.44	**	0.0093	100.85 ± 18.58	ns	0.9999	29.12
AQP6-6	44.50 ± 18.51	*	0.019	92.75 ± 8.81	ns	0.9994	-
AQP6-7 †	165.32 ± 28.59	**	0.0026	86.02 ± 15.78	ns	0.9987	16.90
AQP7-3	44.94 ± 5.52	**	0.004	81.33 ± 9.53	ns	0.9863	80.94
AQP7-4 †	34.14 ± 4.71	***	0.0002	91.65 ± 18.34	ns	0.9993	13.63
AQP7-5 †	36.47 ±7.15	**	0.0038	83.31 ± 7.50	ns	0.9938	31.10
AQP7-6	37.26 ± 5.84	**	0.0045	87.63 ± 25.6	ns	0.999	34.27
AQP8-1	152.87 ± 25.29	*	0.0306	100.23 ± 7.82	ns	0.9999	731.07
AQP8-2 †	100.28 ± 4.578	ns	0.9999	95.20 ± 8.30	ns	0.9996	10.05
AQP8-5 †	86.24 ± 26.82	ns	0.9987	91.08 ± 11.15	ns	0.9993	28.22
AQP8-6	77.03 ± 8.49	ns	0.9159	84.61 ± 12.71	ns	0.9946	84.38
AQP9-1	57.36 ± 10.25	ns	0.0594	83.82 ± 10.36	ns	0.9941	32.76
AQP9-2 †	42.78 ± 13.07	**	0.0024	77.38 ± 10.05	ns	0.9394	18.24
AQP9-4 †	30.81 ± 4.83	****	0.0001	73.84 ± 8.31	ns	0.8285	7.03
AQP9-5	96.40 ± 15.27	ns	0.9997	90.01 ± 7.93	ns	0.9992	23.73
AQP11-4 †	47.59 ± 8.50	*	0.0332	113.19 ± 17.88	ns	0.9988	4.22
AQP11-5 †	99.43 ± 17.82	ns	0.9999	132.52 ± 15.78	ns	0.543	7.80
AQP11-6	89.00 ± 5.98	ns	0.999	78.44 ± 9.11	ns	0.9603	10.26
AQP11-7	80.63 ± 5.57	ns	0.9833	81.26 ± 5.85	ns	0.9861	14.21

† Data for siRNA shown in main text
