# Supplementary material for: Plasmodium parasite exploits host aquaporin-3 during liver stage malaria infection
Source: PLoS Pathog. 2018 May 18;14(5):e1007057. doi: 10.1371/journal.ppat.1007057 (PMC5979039; doi:10.1371/journal.ppat.1007057)
Supplement: S3 Table — (PDF) [file ppat.1007057.s010.pdf]

Table S3. List of gRNAs used for generating AQP3mut cell lineusing the CRISPR/Cas9 genome editing technology.

	gRNA1	gRNA2	Exon Targetted
AQP^{mut1}	caccgAAAGCCAAAGGCCAGGTTGA	caccgACTCTGGGCATCCTCATCGC	2
AQP^{mut2}	caccgGGCGGCGCTGTCGGGCGGGC	GCCATGGGTCGACAGA	1
AQP^{mut3}	caccgAGATGCTCCACATCCGCTACc	×	1
AQP^{mut4}	caccgAAAGCCAAAGGCCAGGTTGA	caccgACTCTGGGCATCCTCATCGC	2
