# Supplementary material for: Plasmodium parasite exploits host aquaporin-3 during liver stage malaria infection
Source: PLoS Pathog. 2018 May 18;14(5):e1007057. doi: 10.1371/journal.ppat.1007057 (PMC5979039; doi:10.1371/journal.ppat.1007057)
Supplement: S4 Table — (PDF) [file ppat.1007057.s011.pdf]

S4 Table. RNA-seq summary data.

ID	Cell line	Time points *	controls	Total Reads	Mapped *H. sapiens* (%)
50040_N_1	HepG2		uninfected	46,351,735	95.3
50043_N_1	HepG2		uninfected	66,947,947	96.3
50045_N_1	HepG2		uninfected	57,453,983	97.1
50046_N_1	HepG2		uninfected	50,403,025	97.2
50039_N_1	HepG2	early		44,059,588	93.1
50044_N_1	HepG2	early		79,005,831	94.7
50048_N_1	HepG2	mid		57,789,037	92.2
50047_N_1	HepG2	mid		60,086,358	78.2
50038_N_1	HepG2	mid		55,843,037	72.5
50042_N_1	HepG2	mid		61,038,757	70.0
50049_N_1	HepG2	late		60,041,207	47.0
50041_N_1	HepG2	late		73,243,131	26.6
50036_N_1	HepG2	late		55,577,831	20.1
50009_N_1	HuH7		uninfected	57,706,211	96.0
50014_N_1	HuH7		uninfected	72,568,339	96.1
50030_N_1	HuH7		uninfected	66,425,265	96.6
50000_N_1	HuH7		uninfected	56,048,512	96.8
50025_N_1	HuH7		uninfected	71,437,692	97.0
50018_N_1	HuH7	early		51,736,339	94.9
50001_N_1	HuH7	early		50,605,071	95.7
50026_N_1	HuH7	early		61,250,321	90.9
50019_N_1	HuH7	early		58,971,982	95.7
50011_N_1	HuH7	early		47,436,418	97.2
50002_N_1	HuH7	early		53,690,301	94.8
50021_N_1	HuH7	early		68,197,956	93.7
50027_N_1	HuH7	early		63,382,872	89.3
50003_N_1	HuH7	early		51,968,890	92.9
50005_N_1	HuH7	early		46,577,577	92.9
50015_N_1	HuH7	mid		62,012,593	95.6
50022_N_1	HuH7	mid		72,154,540	92.7
50013_N_1	HuH7	mid		57,778,178	93.0
50006_N_1	HuH7	mid		60,422,939	91.3
50033_N_1	HuH7	mid		69,486,282	70.8
50023_N_1	HuH7	mid		70,726,532	96.2
50034_N_1	HuH7	late		72,209,410	37.8
50007_N_1	HuH7	late		58,604,057	77.3
50024_N_1	HuH7	late		74,274,913	95.3
50035_N_1	HuH7	late		67,640,317	27.1
50031_N_1	HuH7	late		86,027,190	32.3
50008_N_1	HuH7	late		58,946,943	73.2

*early (2-12 hpi), mid (18-24 hpi), late (36-48 hpi)
